# Randomised controlled trial comparing intraoperative cell salvage and autotransfusion with standard care in the treatment of hip fractures: a protocol for the WHITE 9 study

**DOI:** 10.1136/bmjopen-2022-062338

**Published:** 2022-06-08

**Authors:** Edward Dickenson, Xavier Luke Griffin, Juul Achten, Katy Mironov, Heather O'Connor, Nicholas Parsons, Mike Murphy, Matthew Wyse, James Mason, Duncan Appelbe, Amrita Athwal, Damian Griffin

**Affiliations:** 1 Warwick Medical School, University of Warwick, Coventry, UK; 2 Bone and Joint Health, Barts and The London School of Medicine and Dentistry, Queen Mary University of London, London, UK; 3 Honorary Trauma and Orthopaedic Surgeon, Royal London Hospital, Barts Health NHS Trust, London, UK; 4 Department of Orthopaedics Rheumatology and Musculoskeletal Sciences, University of Oxford, Oxford, UK; 5 NHS Blood and Transplant, John Radcliffe Hospital, Oxford, UK; 6 Consultant Anaesthetist, University Hospitals Coventry and Warwickshire NHS Trust, Coventry, UK

**Keywords:** orthopaedic & trauma surgery, anaesthesia in orthopaedics, hip

## Abstract

**Introduction:**

People who sustain a hip fracture are typically elderly, frail and require urgent surgery. Hip fracture and the urgent surgery is associated with acute blood loss, compounding patients’ pre-existing comorbidities including anaemia. Approximately 30% of patients require a donor blood transfusion in the perioperative period. Donor blood transfusions are associated with increased rates of infections, allergic reactions and longer lengths of stay. Furthermore, there is a substantial cost associated with the use of donor blood. Cell salvage and autotransfusion is a technique that recovers, washes and transfuses blood lost during surgery back to the patient. The objective of this study is to determine the clinical and cost effectiveness of intraoperative cell salvage, compared with standard care, in improving health related quality-of-life of patients undergoing hip fracture surgery.

**Methods and analysis:**

Multicentre, parallel group, two-arm, randomised controlled trial. Patients aged 60 years and older with a hip fracture treated with surgery are eligible. Participants will be randomly allocated on a 1:1 basis to either undergo cell salvage and autotransfusion or they will follow the standard care pathway. Otherwise, all care will be in accordance with the National Institute for Health and Care Excellence guidance. A minimum of 1128 patients will be recruited to obtain 90% power to detect a 0.075-point difference in the primary endpoint: EuroQol-5D-5L HRQoL at 4 months post injury. Secondary outcomes will include complications, postoperative delirium, residential status, mobility, allogenic blood use, mortality and resource use.

**Ethics and dissemination:**

NHS ethical approval was provided on 14 August 2019 (19/WA/0197) and the trial registered (ISRCTN15945622). After the conclusion of this trial, a manuscript will be prepared for peer-review publication. Results will be disseminated in lay form to participants and the public.

**Trial registration number:**

ISRCTN15945622.

**Strengths and limitations of this study**

- Pragmatic multicentre randomised controlled trial.
- Powered to detect differences in health-related quality of life.
- Inclusion of participants with and without cognitive impairment.
- Outcomes include the UK core outcome set for hip fracture.
- The trial will not capture late complications beyond 1 year post surgery.

## Introduction

Sixty-five thousand patients break their hip every year in England, Wales and Northern Ireland.^1^ Globally, the annual incidence was estimated as 1.26 million in 1990 and hip fractures were associated with 740 000 deaths.^2^ Almost all patients with a hip fracture require operative treatment; either internal fixation or arthroplasty in equal numbers.^1^ Despite efforts to rehabilitate these patients, outcomes following surgery are poor; 30-day mortality was 6.5% in 2016, with 1-year mortality estimated to be 30%; furthermore, patients reported a 25% reduction in health-related quality-of-life at 4 months, disability similar to that seen following a stroke.^1 3 4^

Patients admitted with a hip fracture are typically elderly, frail and have multiple medical comorbidities, including prefracture anaemia.^5–8^ As a consequence of the fracture and urgent surgery required, patients sustain acute blood loss, compounding this pre-existing anaemia.^9^ Postoperative anaemia is associated with increased disability, reduced muscle strength and reduced physical performance.^8 10^ Beyond the perioperative period, anaemia is associated with an increased risk of falls, hospitalisation and mortality.^8 11^ In this elderly and frail population perioperative allogenic (blood from a donor) blood transfusion is often required.^12^

Allogenic blood transfusions do not come without risks to patients. They cause an increased rate of local (eg, wound) and systemic (eg, pneumonia) infections in postoperative patients.^13^ This is attributable to the immunomodulatory effect of allogenic blood on the recipient.^13^ As well as causing infections allogenic blood use is independently associated with increased length of hospital stay in orthopaedic surgery.^14^ Rarer direct complications of allogenic blood use include death and major morbidity.^15^

The cost to the NHS of blood replacement products is high; the first unit of red cell concentrates costs £170 with subsequent units costing £162.^16^ At a single major trauma centre, the costs of allogenic blood transfusions for patients with a hip fracture are £62 272 per year (unpublished data). This extrapolates to a direct national cost of approximately £7.28 million. This estimate excludes the costs associated with an increased length of stay and treating infections and other complications of transfusion.

Concerns regarding patient safety and the costs of allogenic blood have driven efforts to reduce transfusion rates.^16^ Intraoperative cell salvage is a method of collecting blood lost during surgery with an option of transfusing it back to the patient. The cell salvage device filters, washes and centrifuges blood lost during surgery, to separate the red blood cells from non-cellular matter prior to intraoperative autotransfusion. Complications as a result of cell salvage are rare.^15^

In order to reduce the use of allogenic blood, the NICE guidelines (Blood Transfusion NG24 2015) recommended the use of cell salvage and tranexamic acid where surgical blood loss is expected to be greater than 500mls.^17^

The direct intraoperative blood loss reported across studies of hip fracture surgery is variable.^18–21^ Several randomised controlled trials report a mean intraoperative blood loss greater than 500 mL in patients undergoing different types of surgery for a fractured hip.^22–25^ When intraoperative losses are added to blood lost as a direct result of the fracture, the total blood loss is estimated to be between 550 ml-1300mL.^9^

When considering whether to use cell salvage, patients with a hip fracture present a unique population. They have a high mortality, high transfusion rates and high degrees of pre-existing morbidity including anaemia. These considerations mean that there are large potential benefits of using cell salvage in this population. Using cell salvage to reduce the use of allogenic blood has the potential benefit to patients of improving their outcomes from hip fracture surgery, by reducing infections, length of stay and levels of anaemia.

It is currently routine practice to use a restrictive transfusion policy in hip fracture surgery, but the use of cell salvage has not become embedded in this patient group. We propose evaluating the clinical and cost effectiveness of cell salvage and autotransfusion in hip fracture surgery.

### Aims and objectives

The aim of this randomised controlled trial is to compare health-related quality of life (HRQoL) in participants over 60 years of age with a surgically treated hip fracture receiving intraoperative cell salvage and autotransfusion, compared with standard care.

The primary objective is:

- To quantify and draw inferences on observed differences in participants’ HRQoL between the trial treatment groups at 4 months post surgery.

The secondary objectives are:

- To quantify and draw inferences on the observed differences in participants’ HRQoL between the trial treatment groups at 12 months post surgery.
- To investigate the risk of complications within the first 12 months post surgery between the trial treatment groups.
- To quantify and draw inferences on observed differences in (1) the proportion of participants suffering with delirium in the immediate postoperative period, (2) residential status at 4 and 12 months post surgery, (3) mobility at 4 and 12 months post surgery, (4) allogenic blood use during the hospital admission and (5) mortality within the first 12 months post surgery between the trial treatment groups.
- To quantify differences in resource use, costs and comparative cost effectiveness of the trial treatment groups in the first year post surgery.

## Methods and analysis

### Study design

A multicentre, parallel group, two-arm, standard-of-care randomised controlled superiority trial assessing the clinical and cost effectiveness of intraoperative cell salvage compared with standard care in patients undergoing surgery for a hip fracture. The trial will be embedded within the World Hip Trauma Evaluation (WHITE) Cohort; a cohort that has delivered a number of embedded RCTs in hip fracture care.^26–29^ The study is conducted in two phases: an initial feasibility phase in which the acceptability of the interventions and trial processes were tested, and a definitive phase which comprises the main trial. Feasibility data will be locked, and not analysed, at completion of that phase. At the end of the definitive main trial phase, data from the two phases will be analysed together as a single dataset.

### Eligibility

Patients will have an eligibility check by the clinical team in the daily trauma meetings. Participants will be assessed against the specific inclusion and exclusion criteria as outlined below:

#### Inclusion criteria

- All patients, both those with and without capacity, presenting with a fracture of the hip (AO type A1-3, B1-3 and subtrochanteric fractures) who, in the opinion of the operating surgeon, would benefit from surgery.

#### Exclusion criteria

- Patients younger than 60 years of age.
- Patients undergoing percutaneous (cannulated) hip screw fixation.
- Patients for whom the treating surgeon has already elected to use cell salvage (eg, a Jehovah’s Witness).
- Patients who have sustained a pathological fracture.

### Consent

Patients with a hip fracture are a clinical priority for urgent operative care. All patients with a fracture of the hip are in pain and will have received opiate analgesia. It is therefore understandable that the majority of patients find the initial period of their treatment in hospital confusing and disorientating. Similarly, patients’ next of kin, carers and friends are often anxious at this time and may have difficulty in absorbing the large amounts of information that they are given about the injury and plan for treatment. In this emergency situation, the focus is on obtaining consent for surgery (where possible) and on informing the patient and any next of kin about immediate clinical care. It is often not possible for the patient, relative or carer (consultee) to review trial documentation, consider the information and communicate an informed decision about whether they would wish to participate in the study. The consent procedure for this trial will reflect that of the surgery, with the clinical team assessing capacity before taking consent for the surgical procedure, and this capacity assessment then being used to guide the proper approach to consenting to the research. An appropriate method, in line with the Mental Capacity Act 2005 and the code of Practice 2007, and approved by the National Research Ethics Committee, will be used to gain either prospective or retrospective consent form the patient or appropriate consultee by a Good Clinical Practice (GCP)-trained, appropriately delegated member of the research team.

### Postrandomisation withdrawals and exclusion

Participants/consultees may withdraw from the study at any time without prejudice. In addition, the investigator may discontinue a participant from the study at any time if the investigator considers it necessary. Throughout the study, screening logs will be kept to determine the number of patients assessed for eligibility and reasons for any exclusion.

If the participant/consultee withdraws from the study completely, data collected from the participant or recorded in the medical record up until the point of withdrawal will be included in the final analysis. Since randomisation will occur just prior to surgery, data regarding the operation received and autotransfusion blood volume (where deemed possible) will be recorded as a minimum for all participants. Participants who decline to continue to take part once they have regained capacity will be given the opportunity to discuss/inform the research team of the reasoning behind their decision not to take part.

Similarly, data from participants who die before consent to continue participating can be obtained, will be included in the final analysis. For those participants who lack capacity, and die before advice can be obtained from the participant’s relatives/next of kin, it is our intention not to contact relatives of participants to inform them of the participant’s initial inclusion in the study to avoid distressing the relatives unnecessarily.

### Randomisation and blinding

The allocation sequence will be generated by the trial statistician. The treatment allocation will be on a 1:1 basis and will be stratified by fracture type (extracapsular vs intracapsular) and by trial centre, to ensure that any clustering effects within centres are evenly distributed between the treatment groups. The allocation will be administered using secure, online randomisation via a distant computer at Oxford Clinical Trials Research Unit (OCTRU), University of Oxford, using RRAMP software. Participants will be randomised preoperatively. The research associate will inform the surgeon and the operating theatre staff of the allocation in the immediate preoperative period.

In order to negate bias in the self-reported HRQoL outcome measures participants will be blinded to treatment allocation. The operating surgeon cannot be blinded to the allocation but they will not be involved in the assessment of outcomes. Participants will be blinded until the completion of the trial when the blinding will be broken if requested by the participants.

### Treatments

#### Preoperative assessments

Diagnosis of a hip fracture will be confirmed by a plain radiograph, as per routine clinical care. Routine investigations, anaesthetic assessment, antibiotic and venous thromboembolic prophylaxis will be used as per local policy.

#### Anaesthetic technique

A regional or general anaesthesia technique will be used for every participant as per routine clinical care. Intraoperative analgesia may be achieved by combining a local anaesthetic nerve block, paracetamol and opiate analgesia as clinically indicated.

#### Trial treatments

All participants will receive perioperative prophylactic antibiotics in accordance with current protocols agreed at each centre. Appropriate preparation, positioning and fracture reduction will be left to the discretion of the operating surgeon, as per their normal clinical practice. The need for allogenic blood products will be determined on an individual patient basis, following each centre’s blood transfusion policy. This will typically involve restrictive transfusion thresholds where asymptomatic patients with a haemoglobin concentration of less than 70 g/L are offered allogenic blood. This threshold may be higher, typically a haemoglobin concentration of less than 80 g/L in those with symptomatic anaemia or coexisting cardiorespiratory disease.

Participants will be randomly allocated to one of the treatment arms:

### Group 1: standard care

A standard suction system removes blood lost in the operating field and it is disposed of in clinical waste.

### Group 2: intraoperative cell salvage and autotransfusion

Intraoperative cell salvage aspirates blood and lavage fluids from the operative field during surgery and returns it to the cell saver device where it is filtered and stored in an Anticoagulant Citrate Dextrose Solution. The recovered fluid will be washed with saline and centrifugated. In all cases where technically sufficient blood is available for transfusion, it will be transferred into a blood-giving bag, where the washed red blood cells, suspended in saline, will be transfused intraoperatively. The volume of blood that was transfused, when this was possible, will be recorded. It will be the responsibility of the treating clinician to ensure that these data are recorded in the clinical notes at the end of surgery. Other relevant information about the operation will be collected.

#### Postoperative rehabilitation

Postoperative analgesia will be prescribed intraoperatively and reviewed by the responsible clinical teams as appropriate. In the postoperative period, as per standard of care, all participants will undergo an initial physiotherapy and occupational therapy trauma assessment. As part of standard care, an initial treatment plan with objectives will be made, recorded and commenced. The aim of this plan will be for participants to mobilise through early, active, full weight bearing.

Participants will be discharged from the acute Orthopaedic Trauma Ward at the earliest safe opportunity to the most appropriate discharge destination as determined by the multidisciplinary clinical team.

### Outcomes

Personal data collected during the study will be handled and stored in accordance with the 2018 Data Protection Act, which requires data to be anonymised as soon as it is practical to do so. The data collected from participants will be entered in linked-anonymised form to the trial database. All electronic patient-identifiable information will be stored on a secure, password-protected database at the University of Oxford, accessible only to the research team.

### Primary outcome measure

The UK Core Outcome Set for hip fracture recommends that patient benefit is best determined by a measure of health-related quality of life.^30 31^ The study primary outcome measure is EuroQol 5 Dimensions 5 Levels (EQ-5D-5L) score at 4 months post injury. EQ-5D-5L is a validated instrument comprising a visual analogue scale (VAS) measuring self-rated health and a health status instrument, consisting of a five-level response (no problems, some problems, moderate problems, severe problems and unable) for five domains related to daily activities^32 33^; (1) mobility, (2) self-care, (3) usual activities, (4) pain and discomfort and (5) anxiety and depression. Responses to the health status classification system will be converted into an overall score using a published utility algorithm for the UK population.^34^ A respondent’s EQ-VAS gives self-rated health on a scale where the endpoints are labelled ‘best imaginable health state’ (100) and ‘worst imaginable health state’ (0). It has been shown to be responsive to change,^31 35^ including when reported by proxy for those with cognitive impairment.^36 37^ Parsons *et al*^38^ modelled patient EQ-5D recovery trajectories after hip fracture surgery to assess the extent of any bias in 4 months outcomes by comparing complete case analysis, model-based projections and data imputation. They showed that imputing a utility of zero for death was a very close approximation to the much more complex projection methods, which was highly dependent on early (pre 4 months) EQ-5D score data that would not be available in the setting of a trial.^38^

### Secondary outcome measures

### Complications

All complications related to the index fracture and its treatment will be recorded. Complications will be classified as:

- related systemic complications^34^ (including venous thromboembolic phenomena, death, pneumonia, urinary tract infection, blood transfusion, acute cerebrovascular incident, acute cardiac event, acute kidney injury, other).
- related local complications (superficial/deep infection, non/mal union, failure/removal/revision of metalwork including further surgery for intraoperative/postoperative periprosthetic fracture, injury to adjacent structures such as nerves/tendons/blood vessels, other).
- unrelated to the trial protocol.

The number and type of related serious adverse events up to 12 months will be recorded.

### Delirium

In line with data collection in the UK NHFD we will collect an immediate preoperative abbreviated mental test score and a postoperative (up to 3 days) 4AT score.

### Residential status

Changes in residential status provide a marker for a participant’s independence through their hip fracture recovery and is one of the recommended core outcomes for trials assessing interventions in hip fractures.^30^ It will be reported by participants or their proxy using an ordinal scale as per the NHFD: (1) own home/sheltered housing, (2) residential care, (3) nursing care, (4) rehabilitation unit—hospital bed in the current trust, (5) rehabilitation unit—hospital bed in another trust, (6) rehabilitation unit—NHS funded care home bed, and (7) acute hospital.

### Mobility

The ability to walk indoors and outdoors is rated very highly by patients.^30^ Mobility will be reported by participants or their proxy using an ordinal scale as per the NHFD: (1) freely mobile without aids, (2) mobile outdoors with one aid, (3) mobile outdoors with two aids or a frame, (4) some indoor mobility but never goes outside without help, and (5) no functional mobility using the lower limbs.

### Units of allogenic blood transfused

The use of allogenic blood products during the index hospital stay will be collected from the trial centres’ blood bank database. For each participant, the number of units transfused and the date of transfusion will be collected.

### Mortality

Mortality during the first 12 months following surgery will be collected from NHS spine (NHS Digital; https://digital.nhs.uk/).

### Resource use

Case report forms will be used to collect resources from medical records during the initial inpatient stay, and post discharge for 12 months at the treating hospital. Further resource use will be collected from the participants to complement the medical records. Participant questionnaires will be administered by telephone or post. They will enquire about hospital contacts related to the index fracture with hospitals other than the index treating sites, rehabilitation units and other care settings. Questions will also ask about the use of equipment and changes to the home, private expenses with rehabilitation services, informal care and loss of productivity.

### Sample size

The sample size for this study is 1128 participants. This full trial sample size is based on the SD of the EQ-5D-5L at 4 months post surgery of 0.3 points^31^ and a minimal clinically important difference of 0.075^39^ with 2-sided significance of 5% requiring 506 with the primary outcome for 80% power or 676 with the primary outcome for 90% power.

In this population, we expect considerable loss to follow-up. Previous WHiTE trials have indicated that these losses are due mainly to patients declining consent to further follow-up, incapacity, and death.^40 41^ We are able to account for participants who have died in our primary outcome measure and have assumed that only 60% of recruited study participants will be available at the definitive endpoint at 4 months.

With a significance level of 5%, this inflates the sample size to 844 for 80% power and 1128 for 90% power. Conservatively, we aim to randomise 1128 in order to ensure a minimum of 676 participants with the primary outcome which will ensure 90% power based on these assumptions.

Similar sample size calculations have been used in existing clinical trials in this patient population (ISRCTN92825709, ISRCTN18393176).

### Statistical analysis

A full, detailed statistical analysis plan (SAP) will be drafted early in the trial and will be finalised following the recruitment review by the Data and Safety Monitoring Committee (DSMC) and Trial Steering Committee (TSC), and prior to the primary analysis data lock. Any subsequent changes to the SAP will be fully justified in the final report.

Baseline demographic data will be summarised to check comparability between treatment arms. Standard statistical summaries and graphical plots will be presented for the primary outcome measure and all secondary outcome measures.

The study analysis will use generalised mixed-effect regression models, with all analyses adjusting for important baseline covariates to improve precision in estimation of the treatment effect. The principal analyses will be conducted on the intention-to-treat population. Differences between intervention arms for the primary outcome measure, EQ-5D-5L^33^ scores at 4 months post surgery, will be analysed by calculating an adjusted treatment effect using a mixed-effect linear regression. A zero value will be imputed for participants who have died prior to this time point. Models will adjust for age, sex, fracture type and cognitive impairment (as fixed effects) and recruitment centre as a random effect to take account of the heterogeneity in the response between centres. The treatment difference will be estimated from the fitted model, together with 95% CIs, with significance set at 5% (2-sided) for comparative tests.

A sensitivity analysis will be performed on a per-protocol (as treated) basis. Further sensitivity analysis of EQ-5D-5L^33^ at 4 months with additional adjustment for the retrospective preinjury baseline EQ-5D-5L^33^ will be carried out to enable the influence of this factor to be evaluated.

Secondary clinical outcomes will be similarly analysed with logistic mixed-effect regression being used for binary data and linear mixed-effect regression for continuous data.

Adverse events will be explored to assess if they differ between groups.

Stata (StatCorp, LP) or other appropriate validated statistical software will be used for all analysis.

### Cost-effectiveness analysis

A within-trial cost-effectiveness analysis will be conducted from the UK NHS and Personal Social Services perspective^42^ in the base case analysis. Resource utilisation involving cost of the cell salvage and autotransfusion if applicable will be obtained from case report forms (CRFs) that will be completed by the local research teams. Broader resource utilisation will be captured through CRFs and patient questionnaires administered at baseline, 4 months, and 12 months post surgery. Unit costs for health and social care resources will largely be derived from the latest available local and national sources and estimated in line with best practice. Costs will be standardised to current prices where appropriate. An incremental cost-effectiveness analysis, expressed in terms of incremental cost per quality-adjusted life year gained, will be performed. Results will be presented using incremental cost-effectiveness ratios, net monetary benefit, and cost effectiveness acceptability curves generated via non-parametric bootstrapping. Multiple imputation methods will be used to impute missing data and avoid biases associated with complete case analysis. Sensitivity analyses involving economic analysis from the societal perspective and extending the time frame from 4 months to 1 year will also be conducted.

### Trial organisation and oversight

The sponsor of this trial is University Hospitals Coventry and Warwickshire NHS trust. The day-to-day management of the trial will be the responsibility of the trial manager, based at the University of Oxford and supported by OCTRU staff. This will be overseen by a trial management group, who will meet monthly to assess progress. It will be the responsibility of the trial manager to undertake training of the research associates at each of the study centres. The study statistician and health economist will be closely involved in setting up data capture systems, design of databases and clinical reporting forms.

A TSC and an independent DAMOCLES^43^ compliant DSMC, that will assess progress, conduct and participant safety, will be set up at the start of the study.

### Quality control

Quality control procedures will be undertaken during recruitment and data collection phases of the study to ensure research is conducted, generated, recorded and reported in compliance with the protocol, GCP and ethics committee. The chief investigators and the trial manager will develop data management and monitoring plans.

### Patient and public involvement

At the centre of this trial is the potential for patient benefit by reducing the risks of hip fracture surgery and improving patient outcomes. The study proposal was discussed with our panel of 15 patient and public members. A member of this panel is a coapplicant on this trial and helped draft the protocol, lay summary and patient information sheet. A lay summary informing patients and the public of the trial outcome will be available on the trial website. Further documentation suitable for the general patient and public communities will be prepared by the research team in collaboration with lay representatives.

### Ethics and dissemination

This study will be embedded within the WHITE portfolio of trials. NHS ethical approval was provided on 14/08/2019 (19/WA/0197) and the trial registered (ISRCTN15945622). The results of this trial will be disseminated to the hip fracture clinical community via presentations at national and international meetings as well as publication in peer-reviewed journal. Results will be disseminated in lay form to participants and the public.

## Supplementary Material

Reviewer comments

Author's manuscript
