# Supplementary material for: Randomised controlled trial comparing intraoperative cell salvage and autotransfusion with standard care in the treatment of hip fractures: a protocol for the WHITE 9 study
Source: BMJ Open. 2022 Jun 8;12(6):e062338. doi: 10.1136/bmjopen-2022-062338 (PMC9185657; doi:10.1136/bmjopen-2022-062338)
Supplement: Author's manuscript [file bmjopen-2022-062338.draft_revisions.pdf]

BMJ Open is committed to open peer review. As part of this commitment we make the peer review history of every article we publish publicly available.

When an article is published we post the peer reviewers' comments and the authors' responses online. We also post the versions of the paper that were used during peer review. These are the versions that the peer review comments apply to.

The versions of the paper that follow are the versions that were submitted during the peer review process. They are not the versions of record or the final published versions. They should not be cited or distributed as the published version of this manuscript.

BMJ Open is an open access journal and the full, final, typeset and author-corrected version of record of the manuscript is available on our site with no access controls, subscription charges or pay-per-view fees (http://bmjopen.bmj.com).

If you have any questions on BMJ Open's open peer review process please email info.bmjopen@bmj.com

BMJ Open

A randomised controlled trial comparing intra-operative cell salvage and autotransfusion with standard care in the treatment of hip fractures: a protocol for the WHITE 9 study

Journal:	*BMJ Open*
Manuscript ID	bmjopen-2022-062338
Article Type:	Protocol
Date Submitted by the Author:	24-Feb-2022

Complete List of Authors:	Dickenson, Edward; University of Warwick, Warwick Medical School<br>Griffin, Xavier; Queen Mary University of London, Trauma & Orthopaedic Surgery;<br>Achten, Juul; University of Oxford, NDORMS<br>Mironov, Katy; University of Oxford Nuffield Department of Orthopaedics Rheumatology and Musculoskeletal Sciences, Oxford Trauma<br>O'Connor , Heather; University of Oxford, NDORMS<br>Parsons, Nicholas; University of Warwick, Warwick Medical School<br>Murphy, Mike; John Radcliffe Hospital, NHS Blood and Transplant<br>Wyse, Matthew; University Hospitals Coventry and Warwickshire NHS Trust<br>Mason, James; University of Warwick, Warwick Medical School<br>Appelbe, Duncan; University of Oxford Nuffield Department of Orthopaedics Rheumatology and Musculoskeletal Sciences<br>Athwal, Amrita; University of Oxford Nuffield Department of Orthopaedics Rheumatology and Musculoskeletal Sciences, Oxford Trauma<br>Griffin, Damian; University of Warwick, Warwick Medical School

Keywords:	Orthopaedic & trauma surgery < SURGERY, Anaesthesia in orthopaedics < ANAESTHETICS, Hip < ORTHOPAEDIC & TRAUMA SURGERY

SCHOLARONE™
Manuscripts

A randomised controlled trial comparing intra-operative cell salvage and autotransfusion with standard care in the treatment of hip fractures: a protocol for the WHITE 9 study

E Dickenson 1, XL Griffin[2,3*], J. Achten[4], K. Mironov[4], H. O'Connor[5], N. Parsons[6], M. Murphy[7, 8, 9], M Wyse [1], J Mason[10], D Appelbe[4], A Athwal[4] and DR Griffin[1,10]

[1] University Hospitals Coventry & Warwickshire NHS Trust, Clifford Bridge Road, Coventry, CV2 2DX

[2] Barts Bone and Joint Health, Barts and The London School of Medicine and Dentistry, 4 Newark Street, London E1 4AT

[3] Barts Health NHS Trust, Royal London Hospital, London

[4] Oxford Trauma and Emergency Care, NDORMS, University of Oxford, Oxford OX3 9DU

[5]Oxford Clinical Trial Unit, NDORMS, University of Oxford, Oxford OX3 9DU, UK

[6] Statistics and epidemiology unit, Warwick Medical School, University of Warwick, Gibbet Hill Road, Coventry, CV4 7AL

[7] NHS Blood and Transplant, 500, North Bristol Park, Filton, Bristol BS34 7QH

[8] Clinical Haematology Department, Oxford University Hospitals NHS Foundation Trust, Oxford, OX3 9DU

[9] Radcliffe Department of medicine, University of Oxford, Oxford, OX3 9DU

[10] Warwick Clinical Trials Unit, Warwick Medical School, University of Warwick, Gibbet Hill Road, Coventry, CV4 7AL, UK

*corresponding author:
Professor Xavier Griffin
Barts Bone and Joint Health, Barts and The London School of Medicine and Dentistry, 4 Newark Street, London E1 4AT
X.griffin@qmul.ac.uk

Main Text Word count 3991

For peer review only - http://bmjopen.bmj.com/site/about/guidelines.xhtml

ABSTRACT

Introduction

People who sustain a hip fracture are typically elderly, frail and require urgent surgery. Hip fracture and the urgent surgery is associated with acute blood loss, compounding patients' pre-existing co-morbidities including anaemia. Approximately 30% of patients require a donor blood transfusion in the perioperative period. Donor blood transfusions are associated with increased rates of infections, allergic reactions and longer lengths of stay. Furthermore, there is a substantial cost associated with the use of donor blood. Cell salvage and autotransfusion is a technique that recovers, washes and transfuses blood lost during surgery back to the patient. The objective of this study is to determine the clinical and cost effectiveness of intraoperative cell salvage, compared to standard care, in improving health related quality-of-life of patients undergoing hip fracture surgery.

Methods and Analysis

Multi-centre, parallel group, two-arm, randomised controlled trial. Patients aged 60 years and older with a hip fracture treated with surgery are eligible. Participants will be randomly allocated on a 1:1 basis to either undergo cell salvage and autotransfusion or they will follow the standard care pathway. Otherwise, all care will be in accordance with the National Institute for Health and Care Excellence guidance. A minimum of 1128 patients will be recruited to obtain 90% power to detect a 0.075-point difference in the primary endpoint: EuroQol-5D-5L HRQoL at 4-months post-injury. Secondary outcomes will include complications, postoperative delirium, residential status, mobility, allogenic blood use, mortality and resource use.

Ethics and Dissemination

NHS ethical approval was provided on 14/08/2019 (19/WA/0197) and the trial registered (ISRCTN15945622). After the conclusion of this trial a manuscript will be prepared for peer review publication. Results will be disseminated in lay form to participants and the public.

Abstract Word Count 280

Keywords:
Hip fracture, Orthopaedic Trauma, Cell Salvage, Auto-transfusion

STRENGTHS & LIMITATIONS

- Pragmatic multi-centre randomised controlled trial
- Powered to detect differences in health-related quality of life
- Inclusion of participants with and without cognitive impairment

For peer review only - http://bmjopen.bmj.com/site/about/guidelines.xhtml

- Outcomes include the UK core outcome set for hip fracture
- The trial will not capture late complications beyond 1-year post surgery

For peer review only

For peer review only - http://bmjopen.bmj.com/site/about/guidelines.xhtml

Introduction

Sixty five thousand patients break their hip every year in England, Wales and Northern Ireland.[1] Globally the annual incidence was estimated as 1.26 million in 1990 and hip fractures were associated with 740,000 deaths.[2] Almost all patients with a hip fracture require operative treatment; either internal fixation or arthroplasty in equal numbers.[1] Despite efforts to rehabilitate these patients, outcomes following surgery are poor; 30-day mortality was 6.5% in 2016, with one-year mortality estimated to be 30%; furthermore, patients reported a 25% reduction in health-related quality-of-life at 4 months, disability similar to that seen following a stroke.[1,3,4]

Patients admitted with a hip fracture are typically elderly, frail and have multiple medical co-morbidities, including pre-fracture anaemia.[5–8] As a consequence of the fracture and urgent surgery required, patients sustain acute blood loss, compounding this pre-existing anaemia.[9] Postoperative anaemia is associated with increased disability, reduced muscle strength and reduced physical performance.[8,10] Beyond the perioperative period, anaemia is associated with an increased risk of falls, hospitalisation and mortality.[8,11] In this elderly and frail population perioperative allogenic (blood from a donor) blood transfusion is often required.[12]

Allogenic blood transfusions do not come without risks to patients. They cause an increased rate of local (e.g. wound) and systemic (e.g. pneumonia) infections in postoperative patients.[13] This is attributable to the immunomodulatory effect of allogenic blood on the recipient.[13] As well as causing infections allogenic blood use is independently associated with increased length of hospital stay in orthopaedic surgery.[14] Rarer direct complications of allogenic blood use include death and major morbidity.[15]

The cost to the NHS of blood replacement products is high; the first unit of red cell concentrates costs £170 with subsequent units costing £162.[16] At a single major trauma centre, the costs of allogenic blood transfusions for patients with a hip fracture are £62,272 per year (unpublished data). This extrapolates to a direct national cost of approximately £7.28 million. This estimate excludes the costs associated with an increased length of stay and treating infections and other complications of transfusion.

Concerns regarding patient safety and the costs of allogenic blood have driven efforts to reduce transfusion rates.[16] Intra operative cell salvage is a method of collecting blood lost during surgery with an option of transfusing it back to the patient. The cell salvage device filters, washes and centrifuges blood lost during surgery, to separate the red blood cells from non-cellular matter prior to intraoperative autotransfusion. Complications as a result of cell salvage are rare.[15]

For peer review only

In order to reduce the use of allogenic blood, the NICE guidelines (Blood Transfusion NG24 2015) recommended the use of cell salvage and tranexamic acid where surgical blood loss is expected to be greater than 500mls.[17]

The direct intra-operative blood loss reported across studies of hip fracture surgery is variable.[18–21] Several randomised controlled trials report a mean intra-operative blood loss greater than 500mls in patients undergoing different types of surgery for a fractured hip.[22–25] When intra operative losses are added to blood lost as a direct result of the fracture, the total blood loss is estimated to be between 550ml-1300ml.[9]

When considering whether to use cell salvage, patients with a hip fracture present a unique population. They have a high mortality, high transfusion rates and high degrees of pre-existing morbidity including anaemia. These considerations mean that there are large potential benefits of using cell salvage in this population. Using cell salvage to reduce the use of allogenic blood has the potential benefit to patients of improving their outcomes from hip fracture surgery, by reducing infections, length of stay and levels of anaemia.

It is currently routine practice to use a restrictive transfusion policy in hip fracture surgery, but the use of cell salvage has not become embedded in this patient group. We propose evaluating the clinical and cost effectiveness of cell salvage and autotransfusion in hip fracture surgery.

For peer review only

Aims and Objectives

The aim of this randomised controlled trial is to compare health-related quality of life (HRQoL) in participants over 60 years of age with a surgically treated hip fracture receiving intraoperative cell salvage and autotransfusion, compared to standard care.

The primary objective is:

- To quantify and draw inferences on observed differences in participants' health-related quality of life between the trial treatment groups at 4 months post-surgery.

The secondary objectives (based on analysis of NHFD data augmented by the UK core outcome set for hip fractures) are:

- To quantify and draw inferences on the observed differences in participants' health-related quality of life between the trial treatment groups at 12 months post-surgery.
- To investigate the risk of complications within the first 12 months post-surgery between the trial treatment groups.
- To quantify and draw inferences on observed differences in (i) the proportion of participants suffering with delirium in the immediate post-operative period, (ii) residential status at 4 and 12 months post-surgery, (iii) mobility at 4 and 12 months post-surgery, (iv) allogenic blood use during the hospital admission and (v) mortality within the first 12 months post-surgery between the trial treatment groups.
- To quantify differences in resource use, costs and comparative cost effectiveness of the trial treatment groups in the first year post-surgery.

For peer review only

METHODS

Study design

A multi-centre, parallel group, two-arm, standard-of-care randomised controlled superiority trial assessing the clinical and cost effectiveness of intraoperative cell salvage compared with standard care in patients undergoing surgery for a hip fracture. The trial will be embedded within the World Hip Trauma Evaluation (WHITE) Cohort; a cohort that has delivered a number of embedded RCTs in hip fracture care.[26–29] The study is conducted in two phases: an initial feasibility phase in which the acceptability of the interventions and trial processes were tested, and a definitive phase which comprises the main trial. Feasibility data will be locked, and not analysed, at completion of that phase. At the end of the definitive main trial phase, data from the two phases will be analysed together as a single dataset.

Eligibility

Patients will have an eligibility check by the clinical team in the daily trauma meetings. Participants will be assessed against the specific inclusion and exclusion criteria as outlined below:

Inclusion criteria

- All patients, both those with and without capacity, presenting with a fracture of the hip (AO type A1-3, B1-3 and sub-trochanteric fractures) who, in the opinion of the operating surgeon, would benefit from surgery.

Exclusion criteria

- Patients younger than 60 years of age.
- Patients undergoing percutaneous (cannulated) hip screw fixation.
- Patients for whom the treating surgeon has already elected to use cell salvage (e.g. a Jehovah Witness).
- Patients who have sustained a pathological fracture.

Consent

Patients with a hip fracture are a clinical priority for urgent operative care. All patients with a fracture of the hip are in pain and will have received opiate analgesia. It is therefore understandable that the majority of patients find the initial period of their treatment in hospital confusing and disorientating. Similarly, patients' next of kin, carers and friends are often anxious at this time and may have difficulty in absorbing the large amounts of information that they are given about the injury and plan for treatment. In this emergency situation the focus is on obtaining consent for surgery (where possible) and on informing the patient and any next of kin about immediate clinical care. It is often not possible for the

For peer review only

patient, relative or carer (consultee) to review trial documentation, consider the information and communicate an informed decision about whether they would wish to participate in the study. The consent procedure for this trial will reflect that of the surgery, with the clinical team assessing capacity before taking consent for the surgical procedure, and this capacity assessment then being used to guide the proper approach to consenting to the research. An appropriate method, in line with the Mental Capacity Act 2005 and the code of Practice 2007, and approved by the National Research Ethics Committee, will be used to gain either prospective or retrospective consent form the patient or appropriate consultee by a Good Clinical Practice (GCP)-trained, appropriately delegated member of the research team.

Post-randomisation withdrawals and exclusion

Participants/consultees may withdraw from the study at any time without prejudice. In addition, the investigator may discontinue a participant from the study at any time if the investigator considers it necessary. Throughout the study, screening logs will be kept to determine the number of patients assessed for eligibility and reasons for any exclusion.

If the participant/consultee withdraws from the study completely, data collected from the participant or recorded in the medical record up until the point of withdrawal will be included in the final analysis. Since randomisation will occur just prior to surgery, data regarding the operation received and autotransfusion blood volume (where deemed possible) will be recorded as a minimum for all participants. Participants who decline to continue to take part once they have regained capacity will be given the opportunity to discuss/inform the research team of the reasoning behind their decision not to take part.

Similarly, data from participants who die before consent to continue participating can be obtained, will be included in the final analysis. For those participants who lack capacity, and die before advice can be obtained from the participant's relatives/next of kin, it is our intention not to contact relatives of participants to inform them of the participant's initial inclusion in the study to avoid distressing the relatives unnecessarily.

Randomisation and blinding

The allocation sequence will be generated by the trial statistician. The treatment allocation will be on a 1:1 basis and will be stratified by fracture type (extracapsular vs intracapsular) and by trial centre, to ensure that any clustering effects within centres are evenly distributed between the treatment groups. The allocation will be administered using secure, online randomisation via a distant computer at Oxford Clinical Trials Research Unit (OCTRU), University of Oxford, using RRAMP software. Participants will be randomised pre-operatively. The research associate will inform the surgeon and the operating theatre staff of the allocation in the immediate pre-operative period.

For peer review only - http://bmjopen.bmj.com/site/about/guidelines.xhtml

In order to negate bias in the self-reported HRQoL outcome measures participants will be blinded to treatment allocation. The operating surgeon cannot be blinded to the allocation but they will not be involved in the assessment of outcomes. Patients will be blinded until the completion of the trial when the blinding will be broken if requested by the participants.

Treatments

Preoperative assessments

Diagnosis of a hip fracture will be confirmed by a plain radiograph, as per routine clinical care. Routine investigations, anaesthetic assessment, antibiotic and venous thromboembolic prophylaxis will be used as per local policy.

Anaesthetic technique

A regional or general anaesthesia technique will be used for every participant as per routine clinical care. Intra-operative analgesia may be achieved by combining a local anaesthetic nerve block, paracetamol and opiate analgesia as clinically indicated.

Trial Treatments

All participants will receive perioperative prophylactic antibiotics in accordance with current protocols agreed at each centre. Appropriate preparation, positioning and fracture reduction will be left to the discretion of the operating surgeon, as per their normal clinical practice. The need for allogenic blood products will be determined on an individual patient basis, following each centre's blood transfusion policy. This will typically involve restrictive transfusion thresholds where asymptomatic patients with a haemoglobin concentration of less than 70g/L are offered allogenic blood. This threshold may be higher, typically a haemoglobin concentration of less than 80g/L in those with symptomatic anaemia or coexisting cardiorespiratory disease.

Participants will be randomly allocated to one of the treatment arms:

Group 1: Standard Care

A standard suction system removes blood lost in the operating field and it is disposed of in clinical waste.

Group 2: Intra operative cell salvage and autotransfusion

Intraoperative cell salvage aspirates blood and lavage fluids from the operative field during surgery and returns it to the cell saver device where it is filtered and stored in an Anticoagulant Citrate Dextrose Solution. The recovered fluid will be washed with saline and centrifugated. In all cases where technically sufficient blood is available for transfusion, it will be transferred into a blood-giving bag, where the washed red blood cells, suspended in saline, will be transfused intraoperatively. The volume of blood that was transfused, when

For peer review only

this was possible, will be recorded. It will be the responsibility of the treating clinician to ensure this data is recorded in the clinical notes at the end of surgery. Other relevant information about the operation will be collected.

Postoperative rehabilitation

Postoperative analgesia will be prescribed intra-operatively and reviewed by the responsible clinical teams as appropriate. In the postoperative period, as per standard of care, all participants will undergo an initial physiotherapy and occupational therapy trauma assessment. As part of standard care, an initial treatment plan with objectives will be made, recorded and commenced. The aim of this plan will be for participants to mobilise through early, active, full weight bearing.

Participants will be discharged from the acute Orthopaedic Trauma Ward at the earliest safe opportunity to the most appropriate discharge destination as determined by the multi-disciplinary clinical team.

Outcomes

Personal data collected during the study will be handled and stored in accordance with the 2018 Data Protection Act, which requires data to be anonymised as soon as it is practical to do so. The data collected from participants will be entered in linked-anonymised form to the trial database. All electronic patient-identifiable information will be stored on a secure, password-protected database at the University of Oxford, accessible only to the research team.

Primary outcome Measure

The UK Core Outcome Set for hip fracture recommends that patient benefit is best determined by a measure of health-related quality-of-life.[30,31] The study primary outcome measure is EuroQol 5 Dimensions 5 Levels (EQ-5D-5L) score at 4-months post-injury. EQ-5D-5L is a validated instrument comprising a visual analogue scale (VAS) measuring self-rated health and a health status instrument, consisting of a five-level response (no problems, some problems, moderate problems, severe problems and unable) for five domains related to daily activities;[32,33] (i) mobility, (ii) self-care, (iii) usual activities, (iv) pain and discomfort and (v) anxiety and depression. Responses to the health status classification system will be converted into an overall score using a published utility algorithm for the UK population.[34] A respondent's EQ-VAS gives self-rated health on a scale where the endpoints are labelled 'best imaginable health state' (100) and 'worst imaginable health state' (0). It has been shown to be responsive to change,[31,35] including when reported by proxy for those with cognitive impairment.[36,37] Parsons et al[38] modelled patient EQ-5D recovery trajectories after hip fracture surgery to assess the extent of any bias in 4 months outcomes by comparing

For peer review only - http://bmjopen.bmj.com/site/about/guidelines.xhtml

complete case analysis, model-based projections and data imputation. They showed that imputing a utility of zero for death was a very close approximation to the much more complex projection methods, which was highly dependent on early (pre 4 months) EQ-5D score data that would not be available in the setting of a trial.[38]

Secondary outcome measures

Complications

All complications related to the index fracture and its treatment will be recorded. Complications will be classified as:

- related systemic complications[34] (including venous thromboembolic phenomena, death, pneumonia, urinary tract infection, blood transfusion, acute cerebrovascular incident, acute cardiac event, acute kidney injury, other).
- related local complications (superficial/deep infection, non/mal union, failure/removal/revision of metalwork including further surgery for intraoperative/ postoperative peri-prosthetic fracture, injury to adjacent structures such as nerves/tendons/blood vessels, other).
- unrelated to the trial protocol.

The number and type of related serious adverse events (SAEs) up to 12 months will be recorded.

Delirium

In line with data collection in the UK National Hip Fracture Database (NHFD) we will collect an immediate pre-operative abbreviated mental test score (AMTS) and a postoperative (up to 3 days) 4AT score.

Residential Status

Changes in residential status provide a marker for a participant's independence through their hip fracture recovery and is one of the recommended core outcomes for trials assessing interventions in hip fractures.[30] It will be reported by participants or their proxy using an ordinal scale as per the NHFD: (1) own home/sheltered housing, (2) residential care, (3) nursing care, (4) rehabilitation unit – hospital bed in the current trust, (5) rehabilitation unit – hospital bed in another trust, (6) rehabilitation unit – NHS funded care home bed, and (7) acute hospital.

Mobility

The ability to walk indoors and outdoors is rated very highly by patients.[30] Mobility will be reported by participants or their proxy using an ordinal scale as per the NHFD: (1) freely mobile without aids, (2) mobile outdoors with one aid, (3) mobile outdoors with two aids or

For peer review only - http://bmjopen.bmj.com/site/about/guidelines.xhtml

a frame, (4) some indoor mobility but never goes outside without help, and (5) no functional mobility using the lower limbs.

Units of allogenic blood transfused

The use of allogenic blood products during the index hospital stay will be collected from the trial centres' blood bank database. For each participant, the number of units transfused and the date of transfusion will be collected.

Mortality

Mortality during the first 12 months following surgery will be collected from NHS spine (NHS Digital; https://digital.nhs.uk/).

Resource use

Case report forms will be used to collect resources from medical records during the initial inpatient stay, and post discharge for 12 months at the treating hospital. Further resource use will be collected from the participants to complement the medical records. Participant questionnaires will be administered by telephone or post. They will enquire about hospital contacts related to the index fracture with hospitals other than the index treating sites, rehabilitation units and other care settings. Questions will also ask about the use of equipment and changes to the home, private expenses with rehabilitation services, informal care and loss of productivity.

Sample size

The sample size for this study is 1128 participants. This full trial sample size is based on the standard deviation of the EQ-5D-5L at 4 months post-surgery of 0.3 points[31] and a minimal clinically important difference of 0.075[39] with 2-sided significance of 5% requiring 506 with the primary outcome for 80% power or 676 with the primary outcome for 90% power.

In this population we expect considerable loss to follow-up. Previous WHiTE trials have indicated that these losses are due mainly to patients declining consent to further follow-up, incapacity, and death.[40,41] We are able to account for participants who have died in our primary outcome measure and have assumed that only 60% of recruited study participants will be available at the definitive endpoint at 4 months. With a significance level of 5%, this inflates the sample size to 844 for 80% power and 1128 for 90% power. Conservatively, we aim to randomise 1128 in order to ensure a minimum of 676 participants with the primary outcome which will ensure 90% power based upon these assumptions.

Similar sample size calculations have been used in existing clinical trials in this patient population (ISRCTN92825709, ISRCTN18393176).

Statistical analysis

A full, detailed Statistical Analysis Plan (SAP) will be drafted early in the trial and will be finalised following the recruitment review by the Data and Safety Monitoring Committee

For peer review only

(DSMC) and Trial Steering Committee (TSC), and prior to the primary analysis data lock. Any subsequent changes to the SAP will be fully justified in the final report.

Baseline demographic data will be summarised to check comparability between treatment arms. Standard statistical summaries and graphical plots will be presented for the primary outcome measure and all secondary outcome measures.

The study analysis will use generalised mixed-effects regression models, with all analyses adjusting for important baseline covariates to improve precision in estimation of the treatment effect. The principal analyses will be conducted on the intention-to-treat (ITT) population. Differences between intervention arms for the primary outcome measure, EQ-5D-5L[33] scores at four months post-surgery, will be analysed by calculating an adjusted treatment effect using a mixed-effects linear regression. A zero value will be imputed for participants who have died prior to this time point. Models will adjust for age, sex, fracture type and cognitive impairment (as fixed effects) and recruitment centre as a random effect to take account of the heterogeneity in the response between centres. The treatment difference will be estimated from the fitted model, together with 95% confidence intervals, with significance set at 5% (2-sided) for comparative tests.

A sensitivity analysis will be performed on a per-protocol (as treated) basis. Further sensitivity analysis of EQ-5D-5L[33] at 4 months with additional adjustment for the retrospective pre-injury baseline EQ-5D-5L[33] will be carried out to enable the influence of this factor to be evaluated.

Secondary clinical outcomes will be similarly analysed with logistic mixed-effects regression being used for binary data and linear mixed-effects regression for continuous data.

Adverse events will be explored to assess if they differ between groups.
Stata (StatCorp, LP) or other appropriate validated statistical software will be used for all analysis.

Cost-effectiveness analysis

A within-trial cost-effectiveness analysis will be conducted from the UK NHS and Personal Social Services perspective (PSS)[42] in the base case analysis. Resource utilisation involving cost of the cell salvage and autotransfusion if applicable will be obtained from case report forms (CRFs) that will be completed by the local research teams. Broader resource utilisation will be captured through CRFs and patient questionnaires administered at baseline, 4 months, and 12 months post-surgery. Unit costs for health and social care resources will largely be derived from the latest available local and national sources and estimated in line with best practice. Costs will be standardised to current prices where appropriate. An incremental cost-effectiveness analysis, expressed in terms of incremental cost per quality-

For peer review only

adjusted life year (QALY) gained, will be performed. Results will be presented using incremental cost-effectiveness ratios (ICERs), net monetary benefit, and cost effectiveness acceptability curves (CEACs) generated via non-parametric bootstrapping. Multiple imputation methods will be used to impute missing data and avoid biases associated with complete case analysis. Sensitivity analyses involving economic analysis from the societal perspective and extending the time frame from four months to one year will also be conducted.

Trial organisation and oversight

The sponsor of this trial is University Hospitals Coventry and Warwickshire NHS trust. The day-to-day management of the trial will be the responsibility of the trial manager, based at the University of Oxford and supported by OCTRU staff. This will be overseen by a trial management group, who will meet monthly to assess progress. It will be the responsibility of the trial manager to undertake training of the research associates at each of the study centres. The study statistician and health economist will be closely involved in setting up data capture systems, design of databases and clinical reporting forms.

A TSC and an independent DAMOCLES[43] compliant DSMC, that will assess progress, conduct and participant safety, will be set up at the start of the study.

Quality control

Quality control procedures will be undertaken during recruitment and data collection phases of the study to ensure research is conducted, generated, recorded and reported in compliance with the protocol, GCP and ethics committee. The chief investigators and the trial manager will develop data management and monitoring plans.

Patient and Public Involvement

At the centre of this trial is the potential for patient benefit by reducing the risks of hip fracture surgery and improving patient outcomes. The study proposal was discussed with our panel of 15 patient and public members. A member of this panel is a co-applicant on this trial and helped draft the protocol, lay summary and patient information sheet. A lay summary informing patients and the public of the trial outcome will be available on the trial website. Further documentation suitable for the general patient and public communities will be prepared by the research team in collaboration with lay representatives.

Dissemination

The results of this trial will be disseminated to the hip fracture clinical community via presentations at national and international meetings as well as publication in peer-reviewed journals.

DISCUSSION

For peer review only - http://bmjopen.bmj.com/site/about/guidelines.xhtml

This study will be embedded within the WHITE portfolio of trials. Part of the methodology described in this protocol, especially the process of informed consent and data collection, has been refined through observations and feedback from stakeholders such as lay representatives, regulatory bodies and recruiting centres involved in other studies within the portfolio.

LIST OF ABBREVIATIONS

AMTS	Abbreviated mental test score
CEAC	Cost Effectiveness Acceptability Curve
COS	Core Outcome Set
CRF	Clinical Reporting Forms
DAMOCLES	Data Monitoring Committees: Lessons, Ethics Statistics Study
DSMC	Data Safety and Monitoring Committee
ICER	incremental cost-effectiveness ratio
ICMJE	International Committee of Medical Journal Editors
EQ-5D	Euroqol- 5 dimensions
GCP	Good Clinical Practice
HRQoL	Health-Related Quality of Life
ITT	Intention to Treat
NHS	National Health Services
NICE	National Institute for Health and Care Excellence
NHFD	National Hip Fracture Database
OCTRU	Oxford Clinical Trials Research Unit
PSS	Personal Social Services
QALY	Quality Adjusted Life Year
RCT	Randomised Clinical Trial
RRAMP	Registration/Randomisation Management Product
SAE	Serious Adverse Event
SAP	Statistical Analysis Plan
TSC	Trial Steering Committee
VAS	Visual Analogue Scale
WHITE	World Hip Trauma Evaluation

DECLARATIONS

Ethics approval

Wales Research Ethics Committee 5 granted ethical approval for the study on 14th August 2019 (19/WA/0197). Participants or an appropriate consultee will provide consent/agreement to enter the study. The present version of the protocol is 5.0 15/7/2020.

For peer review only

Consent to publish

Not applicable

Availability of data and materials

The datasets used and/or analysed during the current study will be available from the corresponding author on reasonable request.

Competing interests

XG is a NIHR Clinician Scientist. James M Mason was a member of the NIHR Health Services and Delivery Research Funding Committee.

Funding

This project is funded by the National Institute for Health Research (NIHR), Research for Patient Benefit (PB-PG-0817-20037 and NIHR202013) and supported by the NIHR Oxford Biomedical Research Centre. The funder did/ will not have a role in study design, collection, analysis, interpretation or writing of the report.

Author Contributors

DG, XG, ED, JA, MW, MM, JM, DA, NP and JR were responsible for obtaining grant funding for this trial. All authors developed the trial protocol and contributed to writing this manuscript. NP and HO developed the statistical analysis plan and are leading the statistical analysis for the study. JM developed the economic analysis plan. All authors reviewed and agreed the final manuscript.

Acknowledgements

Not applicable

Disclaimer

The views expressed in this report are those of the author(s) and not necessarily those of the NIHR, or the Department of Health and Social Care.

For peer review only

REFERENCES

1. National Hip Fracture Database annual report 2016 | RCP London. Accessed November 22, 2021. https://www.rcplondon.ac.uk/projects/outputs/national-hip-fracture-database-annual-report-2016
2. Gullberg B, Johnell O, Kanis JA. World-wide projections for hip fracture. *Osteoporosis international : a journal established as result of cooperation between the European Foundation for Osteoporosis and the National Osteoporosis Foundation of the USA*. 1997;7(5):407-413. doi:10.1007/PL00004148
3. Moran CG, Wenn RT, Sikand M, Taylor AM. Early mortality after hip fracture: is delay before surgery important? *The Journal of bone and joint surgery American volume*. 2005;87(3):483-489. doi:10.2106/JBJS.D.01796
4. Griffin XL, Parsons N, Achten J, Fernandez M, Costa ML. Recovery of health-related quality of life in a United Kingdom hip fracture population. The Warwick Hip Trauma Evaluation--a prospective cohort study. *The bone & joint journal*. 2015;97-B(3):372-382. doi:10.1302/0301-620X.97B3.35738
5. Kenzora JE, Mccarthy RE, J DRENNAN L, Sledge CB. Hip Fracture Mortality: Relation to Age, Treatment, Preopera... : Clinical Orthopaedics and Related Research®. *Clinical orthopaedics and related research 1984; 186: 45-56*. Published online 1984. Accessed November 22, 2021. https://journals.lww.com/clinorthop/Abstract/1984/06000/Hip_Fracture_Mortality__Relation_to_Age,.8.aspx
6. Roche JJW, Wenn RT, Sahota O, Moran CG. Effect of comorbidities and postoperative complications on mortality after hip fracture in elderly people: prospective observational cohort study. *BMJ (Clinical research ed)*. 2005;331(7529):1374-1376. doi:10.1136/BMJ.38643.663843.55
7. Kannegaard PN, van der Mark S, Eiken P, Abrahamsen B. Excess mortality in men compared with women following a hip fracture. National analysis of comedications, comorbidity and survival. *Age and ageing*. 2010;39(2):203-209. doi:10.1093/AGEING/AFP221
8. Penninx BWJH, Pahor M, Woodman RC, Guralnik JM. Anemia in old age is associated with increased mortality and hospitalization. *The journals of gerontology Series A, Biological sciences and medical sciences*. 2006;61(5):474-479. doi:10.1093/GERONA/61.5.474

For peer review only

9. Foss NB, Kehlet H. Hidden blood loss after surgery for hip fracture. *The Journal of bone and joint surgery British volume*. 2006;88(8):1053-1059. doi:10.1302/0301-620X.88B8.17534
10. Foss NB, Kristensen MT, Kehlet H. Anaemia impedes functional mobility after hip fracture surgery. *Age and ageing*. 2008;37(2):173-178. doi:10.1093/AGEING/AFM161
11. Penninx BWJH, Pluijm SMF, Lips P, et al. Late-life anemia is associated with increased risk of recurrent falls. *Journal of the American Geriatrics Society*. 2005;53(12):2106-2111. doi:10.1111/J.1532-5415.2005.00491.X
12. Brunskill SJ, Millette SL, Shokoohi A, et al. Red blood cell transfusion for people undergoing hip fracture surgery. *The Cochrane database of systematic reviews*. 2015;2015(4). doi:10.1002/14651858.CD009699.PUB2
13. Rohde JM, Dimcheff DE, Blumberg N, et al. Health care-associated infection after red blood cell transfusion: a systematic review and meta-analysis. *JAMA*. 2014;311(13):1317-1326. doi:10.1001/JAMA.2014.2726
14. Bou Monsef J, Boettner F. Blood management may have an impact on length of stay after total hip arthroplasty. *HSS journal : the musculoskeletal journal of Hospital for Special Surgery*. 2014;10(2):124-130. doi:10.1007/S11420-014-9384-X
15. Bolton-Maggs P, Poles D, Group eaobotSHoTSS. The 2016 annual SHOT Report, 2017. Accessed November 22, 2021. https://www.shotuk.org/wp-content/uploads/myimages/SHOT-Report-2016_web_11th-July.pdf
16. Excellence NIoHaC. Costing Statement | Blood transfusion | Guidance | NICE. Published 2015. Accessed November 22, 2021. https://www.nice.org.uk/guidance/NG24/resources
17. Excellence NIoHaC. Blood transfusion | Guidance | NICE. Published 2015. Accessed November 22, 2021. https://www.nice.org.uk/guidance/ng24
18. Parker MJ, Gurusamy KS. Internal fixation versus arthroplasty for intracapsular proximal femoral fractures in adults. *The Cochrane database of systematic reviews*. 2006;2006(4). doi:10.1002/14651858.CD001708.PUB2
19. Parker MJ, Handoll HHG. Replacement arthroplasty versus internal fixation for extracapsular hip fractures in adults. *The Cochrane database of systematic reviews*. 2006;2006(2). doi:10.1002/14651858.CD000086.PUB2
20. Queally JM, Harris E, Handoll HHG, Parker MJ. Intramedullary nails for extracapsular hip fractures in adults. *The Cochrane database of systematic reviews*. 2014;2014(9). doi:10.1002/14651858.CD004961.PUB4
21. Parker MJ, Das A. Extramedullary fixation implants and external fixators for extracapsular hip fractures in adults. *The Cochrane database of systematic reviews*. 2013;2013(2). doi:10.1002/14651858.CD000339.PUB3
22. Kim SY, Kim YG, Hwang JK. Cementless calcar-replacement hemiarthroplasty compared with intramedullary fixation of unstable intertrochanteric fractures. A prospective,

For peer review only

randomized study. *The Journal of bone and joint surgery American volume*. 2005;87(10):2186-2192. doi:10.2106/JBJS.D.02768

23. Vossinakis IC, Badras LS. The external fixator compared with the sliding hip screw for pertrochanteric fractures of the femur. *The Journal of bone and joint surgery British volume*. 2002;84(1):23-29. doi:10.1302/0301-620X.84B1.12034
24. Rödén M, Schön M, Fredin H. Treatment of displaced femoral neck fractures: a randomized minimum 5-year follow-up study of screws and bipolar hemiprostheses in 100 patients. *Acta orthopaedica Scandinavica*. 2003;74(1):42-44. doi:10.1080/00016470310013635
25. Tidermark J, Ponzer S, Svensson O, Söderqvist A, Törnkvist H. Internal fixation compared with total hip replacement for displaced femoral neck fractures in the elderly. A randomised, controlled trial. *The Journal of bone and joint surgery British volume*. 2003;85(3):380-388. doi:10.1302/0301-620X.85B3.13609
26. Costa ML, Griffin XL, Achten J, et al. World Hip Trauma Evaluation (WHiTE): framework for embedded comprehensive cohort studies. *BMJ open*. 2016;6(10). doi:10.1136/BMJOPEN-2016-011679
27. Griffin XL, McArthur J, Achten J, Parsons N, Costa ML. The Warwick Hip Trauma Evaluation One -an abridged protocol for the WHiTE One Study: An embedded randomised trial comparing the X-bolt with slidinghip screw fixation in extracapsular hip fractures. *Bone & joint research*. 2013;2(10):206-209. doi:10.1302/2046-3758.210.2000183
28. Huxley C, Achten J, Costa ML, Griffiths F, Griffin XL. A process evaluation of the WHiTE Two trial comparing total hip arthroplasty with and without dual mobility component in the treatment of displaced intracapsular fractures of the proximal femur: Can a trial investigating total hip arthroplasty for hip fracture be delivered in the NHS? *Bone & joint research*. 2016;5(10):444-452. doi:10.1302/2046-3758.510.BJR-2015-0008.R1
29. Sims AL, Parsons N, Achten J, Griffin XL, Costa ML, Reed MR. The World Hip Trauma Evaluation Study 3: Hemiarthroplasty Evaluation by Multicentre Investigation - WHITE 3: HEMI - An Abridged Protocol. *Bone & joint research*. 2016;5(1):18-25. doi:10.1302/2046-3758.51.2000473
30. Haywood KL, Griffin XL, Achten J, Costa ML. Developing a core outcome set for hip fracture trials. *The bone & joint journal*. 2014;96-B(8):1016-1023. doi:10.1302/0301-620X.96B8.33766
31. Parsons N, Griffin XL, Achten J, Costa ML. Outcome assessment after hip fracture: is EQ-5D the answer? *Bone & joint research*. 2014;3(3):69-75. doi:10.1302/2046-3758.33.2000250
32. Brooks R, de Charro F. EuroQol: the current state of play. *Health policy (Amsterdam, Netherlands)*. 1996;37(1):53-72. doi:10.1016/0168-8510(96)00822-6
33. Herdman M, Gudex C, Lloyd A, et al. Development and preliminary testing of the new five-level version of EQ-5D (EQ-5D-5L). *Quality of life research : an international journal*

For peer review only

of quality of life aspects of treatment, care and rehabilitation. 2011;20(10):1727-1736. doi:10.1007/S11136-011-9903-X

34. Dolan P. Modeling valuations for EuroQol health states. *Medical care*. 1997;35(11):1095-1108. doi:10.1097/00005650-199711000-00002

35. Tidermark J, Bergström G. Responsiveness of the EuroQol (EQ-5D) and the Nottingham Health Profile (NHP) in elderly patients with femoral neck fractures. *Quality of life research : an international journal of quality of life aspects of treatment, care and rehabilitation*. 2007;16(2):321-330. doi:10.1007/S11136-006-9004-4

36. Bryan S, Hardyman W, Bentham P, Buckley A, Laight A. Proxy completion of EQ-5D in patients with dementia. *Quality of life research : an international journal of quality of life aspects of treatment, care and rehabilitation*. 2005;14(1):107-118. doi:10.1007/S11136-004-1920-6

37. Hounsome N, Orrell M, Edwards RT. EQ-5D as a quality of life measure in people with dementia and their carers: evidence and key issues. *Value in health : the journal of the International Society for Pharmacoeconomics and Outcomes Research*. 2011;14(2):390-399. doi:10.1016/J.JVAL.2010.08.002

38. Parsons N, Griffin XL, Achten J, Chesser TJ, Lamb SE, Costa ML. Modelling and estimation of health-related quality of life after hip fracture: A re-analysis of data from a prospective cohort study. *Bone & joint research*. 2018;7(1):1-5. doi:10.1302/2046-3758.71.BJR-2017-0199

39. Walters SJ, Brazier JE. Comparison of the minimally important difference for two health state utility measures: EQ-5D and SF-6D. *Quality of life research : an international journal of quality of life aspects of treatment, care and rehabilitation*. 2005;14(6):1523-1532. doi:10.1007/S11136-004-7713-0

40. Sims AL, Parsons N, Achten J, Griffin XL, Costa ML, Reed MR. A randomized controlled trial comparing the Thompson hemiarthroplasty with the Exeter polished tapered stem and Unitrax modular head in the treatment of displaced intracapsular fractures of the hip: the WHiTE 3: HEMI Trial. *The bone & joint journal*. 2018;100-B(3):352-360. doi:10.1302/0301-620X.100B3.BJJ-2017-0872.R2

41. Griffin XL, Achten J, Sones W, Cook J, Costa ML. Randomised controlled trial of the sliding hip screw versus X-Bolt Dynamic Hip Plating System for the fixation of trochanteric fractures of the hip in adults: a protocol study for WHiTE 4 (WHiTE4). *BMJ open*. 2018;8(1). doi:10.1136/BMJOPEN-2017-019944

42. Guide to the Methods of Technology Appraisal 2013. London. Accessed November 22, 2021. https://pubmed.ncbi.nlm.nih.gov/27905712/

43. A proposed charter for clinical trial data monitoring committees: helping them to do their job well. *Lancet (London, England)*. 2005;365(9460):711-722. doi:10.1016/S0140-6736(05)17965-3

For peer review only

For peer review only

For peer review only - http://bmjopen.bmj.com/site/about/guidelines.xhtml

SPIRIT

STANDARD PROTOCOL ITEMS: RECOMMENDATIONS FOR INTERVENTIONAL TRIALS

SPIRIT 2013 Checklist: Recommended items to address in a clinical trial protocol and related documents*

Section/item	Item No	Description	Page/ Location
**Administrative information**			
Title	1	Descriptive title identifying the study design, population, interventions, and, if applicable, trial acronym	1
Trial registration	2a	Trial identifier and registry name. If not yet registered, name of intended registry	2
	2b	All items from the World Health Organization Trial Registration Data Set	2
Protocol version	3	Date and version identifier	15
Funding	4	Sources and types of financial, material, and other support	16
Roles and responsibilities	5a	Names, affiliations, and roles of protocol contributors	16
	5b	Name and contact information for the trial sponsor	14
	5c	Role of study sponsor and funders, if any, in study design; collection, management, analysis, and interpretation of data; writing of the report; and the decision to submit the report for publication, including whether they will have ultimate authority over any of these activities	16
	5d	Composition, roles, and responsibilities of the coordinating centre, steering committee, endpoint adjudication committee, data management team, and other individuals or groups overseeing the trial, if applicable (see Item 21a for data monitoring committee)	14

For peer review only

**Introduction**			
Background and rationale	6a	Description of research question and justification for undertaking the trial, including summary of relevant studies (published and unpublished) examining benefits and harms for each intervention	4
	6b	Explanation for choice of comparators	4
Objectives	7	Specific objectives or hypotheses	6
Trial design	8	Description of trial design including type of trial (eg, parallel group, crossover, factorial, single group), allocation ratio, and framework (eg, superiority, equivalence, noninferiority, exploratory)	7
**Methods: Participants, interventions, and outcomes**			
Study setting	9	Description of study settings (eg, community clinic, academic hospital) and list of countries where data will be collected. Reference to where list of study sites can be obtained	7
Eligibility criteria	10	Inclusion and exclusion criteria for participants. If applicable, eligibility criteria for study centres and individuals who will perform the interventions (eg, surgeons, psychotherapists)	7
Interventions	11a	Interventions for each group with sufficient detail to allow replication, including how and when they will be administered	9
	11b	Criteria for discontinuing or modifying allocated interventions for a given trial participant (eg, drug dose change in response to harms, participant request, or improving/worsening disease)	7-9
	11c	Strategies to improve adherence to intervention protocols, and any procedures for monitoring adherence (eg, drug tablet return, laboratory tests)	7-9
	11d	Relevant concomitant care and interventions that are permitted or prohibited during the trial	9

For peer review only - http://bmjopen.bmj.com/site/about/guidelines.xhtml

Outcomes	12	Primary, secondary, and other outcomes, including the specific measurement variable (eg, systolic blood pressure), analysis metric (eg, change from baseline, final value, time to event), method of aggregation (eg, median, proportion), and time point for each outcome. Explanation of the clinical relevance of chosen efficacy and harm outcomes is strongly recommended	10-12
Participant timeline	13	Time schedule of enrolment, interventions (including any run-ins and washouts), assessments, and visits for participants. A schematic diagram is highly recommended (see Figure)	7-12
Sample size	14	Estimated number of participants needed to achieve study objectives and how it was determined, including clinical and statistical assumptions supporting any sample size calculations	12
Recruitment	15	Strategies for achieving adequate participant enrolment to reach target sample size	7-8
**Methods: Assignment of interventions (for controlled trials)**			
Allocation:			
Sequence generation	16a	Method of generating the allocation sequence (eg, computer-generated random numbers), and list of any factors for stratification. To reduce predictability of a random sequence, details of any planned restriction (eg, blocking) should be provided in a separate document that is unavailable to those who enrol participants or assign interventions	8-9
Allocation concealment mechanism	16b	Mechanism of implementing the allocation sequence (eg, central telephone; sequentially numbered, opaque, sealed envelopes), describing any steps to conceal the sequence until interventions are assigned	8-9
Implementation	16c	Who will generate the allocation sequence, who will enrol participants, and who will assign participants to interventions	8-9
Blinding (masking)	17a	Who will be blinded after assignment to interventions (eg, trial participants, care providers, outcome assessors, data analysts), and how	9

	17b	If blinded, circumstances under which unblinding is permissible, and procedure for revealing a participant's allocated intervention during the trial	9
**Methods: Data collection, management, and analysis**			
Data collection methods	18a	Plans for assessment and collection of outcome, baseline, and other trial data, including any related processes to promote data quality (eg, duplicate measurements, training of assessors) and a description of study instruments (eg, questionnaires, laboratory tests) along with their reliability and validity, if known. Reference to where data collection forms can be found, if not in the protocol	10-12
	18b	Plans to promote participant retention and complete follow-up, including list of any outcome data to be collected for participants who discontinue or deviate from intervention protocols	7-8
Data management	19	Plans for data entry, coding, security, and storage, including any related processes to promote data quality (eg, double data entry; range checks for data values). Reference to where details of data management procedures can be found, if not in the protocol	10
Statistical methods	20a	Statistical methods for analysing primary and secondary outcomes. Reference to where other details of the statistical analysis plan can be found, if not in the protocol	12-14
	20b	Methods for any additional analyses (eg, subgroup and adjusted analyses)	12-14
	20c	Definition of analysis population relating to protocol non-adherence (eg, as randomised analysis), and any statistical methods to handle missing data (eg, multiple imputation)	12-14
**Methods: Monitoring**			
Data monitoring	21a	Composition of data monitoring committee (DMC); summary of its role and reporting structure; statement of whether it is independent from the sponsor and competing interests; and reference to where further details about its charter can be found, if not in the protocol. Alternatively, an explanation of why a DMC is not needed	14

	21b	Description of any interim analyses and stopping guidelines, including who will have access to these interim results and make the final decision to terminate the trial	12-14
Harms	22	Plans for collecting, assessing, reporting, and managing solicited and spontaneously reported adverse events and other unintended effects of trial interventions or trial conduct	11
Auditing	23	Frequency and procedures for auditing trial conduct, if any, and whether the process will be independent from investigators and the sponsor	14
**Ethics and dissemination**			
Research ethics approval	24	Plans for seeking research ethics committee/institutional review board (REC/IRB) approval	15
Protocol amendments	25	Plans for communicating important protocol modifications (eg, changes to eligibility criteria, outcomes, analyses) to relevant parties (eg, investigators, REC/IRBs, trial participants, trial registries, journals, regulators)	15
Consent or assent	26a	Who will obtain informed consent or assent from potential trial participants or authorised surrogates, and how (see Item 32)	7-8
	26b	Additional consent provisions for collection and use of participant data and biological specimens in ancillary studies, if applicable	n/a
Confidentiality	27	How personal information about potential and enrolled participants will be collected, shared, and maintained in order to protect confidentiality before, during, and after the trial	10
Declaration of interests	28	Financial and other competing interests for principal investigators for the overall trial and each study site	16
Access to data	29	Statement of who will have access to the final trial dataset, and disclosure of contractual agreements that limit such access for investigators	12

For peer review only

Ancillary and post-trial care	30	Provisions, if any, for ancillary and post-trial care, and for compensation to those who suffer harm from trial participation	n/a
Dissemination policy	31a	Plans for investigators and sponsor to communicate trial results to participants, healthcare professionals, the public, and other relevant groups (eg, via publication, reporting in results databases, or other data sharing arrangements), including any publication restrictions	14
	31b	Authorship eligibility guidelines and any intended use of professional writers	16
	31c	Plans, if any, for granting public access to the full protocol, participant-level dataset, and statistical code	12
**Appendices**			
Informed consent materials	32	Model consent form and other related documentation given to participants and authorised surrogates	n/a
Biological specimens	33	Plans for collection, laboratory evaluation, and storage of biological specimens for genetic or molecular analysis in the current trial and for future use in ancillary studies, if applicable	n/a

*It is strongly recommended that this checklist be read in conjunction with the SPIRIT 2013 Explanation & Elaboration for important clarification on the items. Amendments to the protocol should be tracked and dated. The SPIRIT checklist is copyrighted by the SPIRIT Group under the Creative Commons "Attribution-NonCommercial-NoDerivs 3.0 Unported" license.

For peer review only

For peer review only - http://bmjopen.bmj.com/site/about/guidelines.xhtml

BMJ Open

A randomised controlled trial comparing intra-operative cell salvage and autotransfusion with standard care in the treatment of hip fractures: a protocol for the WHITE 9 study

Journal:	*BMJ Open*
Manuscript ID	bmjopen-2022-062338.R1
Article Type:	Protocol
Date Submitted by the Author:	29-Mar-2022
Complete List of Authors:	Dickenson, Edward; University of Warwick, Warwick Medical School<br>Griffin, Xavier; Queen Mary University of London, Trauma & Orthopaedic Surgery;<br>Achten, Juul; University of Oxford, NDORMS<br>Mironov, Katy; University of Oxford Nuffield Department of Orthopaedics Rheumatology and Musculoskeletal Sciences, Oxford Trauma<br>O'Connor , Heather; University of Oxford, NDORMS<br>Parsons, Nicholas; University of Warwick, Warwick Medical School<br>Murphy, Mike; John Radcliffe Hospital, NHS Blood and Transplant<br>Wyse, Matthew; University Hospitals Coventry and Warwickshire NHS Trust<br>Mason, James; University of Warwick, Warwick Medical School<br>Appelbe, Duncan; University of Oxford Nuffield Department of Orthopaedics Rheumatology and Musculoskeletal Sciences<br>Athwal, Amrita; University of Oxford Nuffield Department of Orthopaedics Rheumatology and Musculoskeletal Sciences, Oxford Trauma<br>Griffin, Damian; University of Warwick, Warwick Medical School
**Primary Subject Heading**:	Surgery
Secondary Subject Heading:	Anaesthesia, Surgery, Haematology (incl blood transfusion)
Keywords:	Orthopaedic & trauma surgery < SURGERY, Anaesthesia in orthopaedics < ANAESTHETICS, Hip < ORTHOPAEDIC & TRAUMA SURGERY

SCHOLARONE™
Manuscripts

A randomised controlled trial comparing intra-operative cell salvage and autotransfusion with standard care in the treatment of hip fractures: a protocol for the WHITE 9 study

E Dickenson 1, XL Griffin[2,3*], J. Achten[4], K. Mironov[4], H. O'Connor[5], N. Parsons[6], M. Murphy[7, 8, 9], M Wyse [1], J Mason[10], D Appelbe[4], A Athwal[4] and DR Griffin[1,10]

[1] University Hospitals Coventry & Warwickshire NHS Trust, Clifford Bridge Road, Coventry, CV2 2DX

[2] Barts Bone and Joint Health, Barts and The London School of Medicine and Dentistry, 4 Newark Street, London E1 4AT

[3] Barts Health NHS Trust, Royal London Hospital, London

[4] Oxford Trauma and Emergency Care, NDORMS, University of Oxford, Oxford OX3 9DU

[5] Oxford Clinical Trial Unit, NDORMS, University of Oxford, Oxford OX3 9DU, UK

[6] Statistics and epidemiology unit, Warwick Medical School, University of Warwick, Gibbet Hill Road, Coventry, CV4 7AL

[7] NHS Blood and Transplant, 500, North Bristol Park, Filton, Bristol BS34 7QH

[8] Clinical Haematology Department, Oxford University Hospitals NHS Foundation Trust, Oxford, OX3 9DU

[9] Radcliffe Department of medicine, University of Oxford, Oxford, OX3 9DU

[10] Warwick Clinical Trials Unit, Warwick Medical School, University of Warwick, Gibbet Hill Road, Coventry, CV4 7AL, UK

*corresponding author:
Professor Xavier Griffin
Barts Bone and Joint Health, Barts and The London School of Medicine and Dentistry, 4 Newark Street, London E1 4AT
X.griffin@qmul.ac.uk

Main Text Word count 3991

For peer review only

ABSTRACT

Introduction

People who sustain a hip fracture are typically elderly, frail and require urgent surgery. Hip fracture and the urgent surgery is associated with acute blood loss, compounding patients' pre-existing co-morbidities including anaemia. Approximately 30% of patients require a donor blood transfusion in the perioperative period. Donor blood transfusions are associated with increased rates of infections, allergic reactions and longer lengths of stay. Furthermore, there is a substantial cost associated with the use of donor blood. Cell salvage and autotransfusion is a technique that recovers, washes and transfuses blood lost during surgery back to the patient. The objective of this study is to determine the clinical and cost effectiveness of intraoperative cell salvage, compared to standard care, in improving health related quality-of-life of patients undergoing hip fracture surgery.

Methods and Analysis

Multi-centre, parallel group, two-arm, randomised controlled trial. Patients aged 60 years and older with a hip fracture treated with surgery are eligible. Participants will be randomly allocated on a 1:1 basis to either undergo cell salvage and autotransfusion or they will follow the standard care pathway. Otherwise, all care will be in accordance with the National Institute for Health and Care Excellence guidance. A minimum of 1128 patients will be recruited to obtain 90% power to detect a 0.075-point difference in the primary endpoint: EuroQol-5D-5L HRQoL at 4-months post-injury. Secondary outcomes will include complications, postoperative delirium, residential status, mobility, allogenic blood use, mortality and resource use.

Ethics and Dissemination

NHS ethical approval was provided on 14/08/2019 (19/WA/0197) and the trial registered (ISRCTN15945622). After the conclusion of this trial a manuscript will be prepared for peer review publication. Results will be disseminated in lay form to participants and the public.

Abstract Word Count 280

Keywords:
Hip fracture, Orthopaedic Trauma, Cell Salvage, Auto-transfusion

STRENGTHS & LIMITATIONS

- Pragmatic multi-centre randomised controlled trial
- Powered to detect differences in health-related quality of life
- Inclusion of participants with and without cognitive impairment

For peer review only - http://bmjopen.bmj.com/site/about/guidelines.xhtml

- Outcomes include the UK core outcome set for hip fracture
- The trial will not capture late complications beyond 1-year post surgery

For peer review only

For peer review only - http://bmjopen.bmj.com/site/about/guidelines.xhtml

INTRODUCTION

Sixty five thousand patients break their hip every year in England, Wales and Northern Ireland.[1] Globally the annual incidence was estimated as 1.26 million in 1990 and hip fractures were associated with 740,000 deaths.[2] Almost all patients with a hip fracture require operative treatment; either internal fixation or arthroplasty in equal numbers.[1] Despite efforts to rehabilitate these patients, outcomes following surgery are poor; 30-day mortality was 6.5% in 2016, with one-year mortality estimated to be 30%; furthermore, patients reported a 25% reduction in health-related quality-of-life at 4 months, disability similar to that seen following a stroke.[1,3,4]

Patients admitted with a hip fracture are typically elderly, frail and have multiple medical co-morbidities, including pre-fracture anaemia.[5–8] As a consequence of the fracture and urgent surgery required, patients sustain acute blood loss, compounding this pre-existing anaemia.[9] Postoperative anaemia is associated with increased disability, reduced muscle strength and reduced physical performance.[8,10] Beyond the perioperative period, anaemia is associated with an increased risk of falls, hospitalisation and mortality.[8,11] In this elderly and frail population perioperative allogenic (blood from a donor) blood transfusion is often required.[12]

Allogenic blood transfusions do not come without risks to patients. They cause an increased rate of local (e.g. wound) and systemic (e.g. pneumonia) infections in postoperative patients.[13] This is attributable to the immunomodulatory effect of allogenic blood on the recipient.[13] As well as causing infections allogenic blood use is independently associated with increased length of hospital stay in orthopaedic surgery.[14] Rarer direct complications of allogenic blood use include death and major morbidity.[15]

The cost to the NHS of blood replacement products is high; the first unit of red cell concentrates costs £170 with subsequent units costing £162.[16] At a single major trauma centre, the costs of allogenic blood transfusions for patients with a hip fracture are £62,272 per year (unpublished data). This extrapolates to a direct national cost of approximately £7.28 million. This estimate excludes the costs associated with an increased length of stay and treating infections and other complications of transfusion.

Concerns regarding patient safety and the costs of allogenic blood have driven efforts to reduce transfusion rates.[16] Intra operative cell salvage is a method of collecting blood lost during surgery with an option of transfusing it back to the patient. The cell salvage device filters, washes and centrifuges blood lost during surgery, to separate the red blood cells from non-cellular matter prior to intraoperative autotransfusion. Complications as a result of cell salvage are rare.[15]

For peer review only - http://bmjopen.bmj.com/site/about/guidelines.xhtml

In order to reduce the use of allogenic blood, the NICE guidelines (Blood Transfusion NG24 2015) recommended the use of cell salvage and tranexamic acid where surgical blood loss is expected to be greater than 500mls.[17]

The direct intra-operative blood loss reported across studies of hip fracture surgery is variable.[18–21] Several randomised controlled trials report a mean intra-operative blood loss greater than 500mls in patients undergoing different types of surgery for a fractured hip.[22–25] When intra operative losses are added to blood lost as a direct result of the fracture, the total blood loss is estimated to be between 550ml-1300ml.[9]

When considering whether to use cell salvage, patients with a hip fracture present a unique population. They have a high mortality, high transfusion rates and high degrees of pre-existing morbidity including anaemia. These considerations mean that there are large potential benefits of using cell salvage in this population. Using cell salvage to reduce the use of allogenic blood has the potential benefit to patients of improving their outcomes from hip fracture surgery, by reducing infections, length of stay and levels of anaemia.

It is currently routine practice to use a restrictive transfusion policy in hip fracture surgery, but the use of cell salvage has not become embedded in this patient group. We propose evaluating the clinical and cost effectiveness of cell salvage and autotransfusion in hip fracture surgery.

For peer review only

Aims and Objectives

The aim of this randomised controlled trial is to compare health-related quality of life (HRQoL) in participants over 60 years of age with a surgically treated hip fracture receiving intraoperative cell salvage and autotransfusion, compared to standard care.

The primary objective is:

- To quantify and draw inferences on observed differences in participants' health-related quality of life between the trial treatment groups at 4 months post-surgery.

The secondary objectives (based on analysis of NHFD data augmented by the UK core outcome set for hip fractures) are:

- To quantify and draw inferences on the observed differences in participants' health-related quality of life between the trial treatment groups at 12 months post-surgery.
- To investigate the risk of complications within the first 12 months post-surgery between the trial treatment groups.
- To quantify and draw inferences on observed differences in (i) the proportion of participants suffering with delirium in the immediate post-operative period, (ii) residential status at 4 and 12 months post-surgery, (iii) mobility at 4 and 12 months post-surgery, (iv) allogenic blood use during the hospital admission and (v) mortality within the first 12 months post-surgery between the trial treatment groups.
- To quantify differences in resource use, costs and comparative cost effectiveness of the trial treatment groups in the first year post-surgery.

For peer review only

METHODS AND ANALYSIS

Study design

A multi-centre, parallel group, two-arm, standard-of-care randomised controlled superiority trial assessing the clinical and cost effectiveness of intraoperative cell salvage compared with standard care in patients undergoing surgery for a hip fracture. The trial will be embedded within the World Hip Trauma Evaluation (WHITE) Cohort; a cohort that has delivered a number of embedded RCTs in hip fracture care.[26–29] The study is conducted in two phases: an initial feasibility phase in which the acceptability of the interventions and trial processes were tested, and a definitive phase which comprises the main trial. Feasibility data will be locked, and not analysed, at completion of that phase. At the end of the definitive main trial phase, data from the two phases will be analysed together as a single dataset.

Eligibility

Patients will have an eligibility check by the clinical team in the daily trauma meetings. Participants will be assessed against the specific inclusion and exclusion criteria as outlined below:

Inclusion criteria

- All patients, both those with and without capacity, presenting with a fracture of the hip (AO type A1-3, B1-3 and sub-trochanteric fractures) who, in the opinion of the operating surgeon, would benefit from surgery.

Exclusion criteria

- Patients younger than 60 years of age.
- Patients undergoing percutaneous (cannulated) hip screw fixation.
- Patients for whom the treating surgeon has already elected to use cell salvage (e.g. a Jehovah Witness).
- Patients who have sustained a pathological fracture.

Consent

Patients with a hip fracture are a clinical priority for urgent operative care. All patients with a fracture of the hip are in pain and will have received opiate analgesia. It is therefore understandable that the majority of patients find the initial period of their treatment in hospital confusing and disorientating. Similarly, patients' next of kin, carers and friends are often anxious at this time and may have difficulty in absorbing the large amounts of information that they are given about the injury and plan for treatment. In this emergency situation the focus is on obtaining consent for surgery (where possible) and on informing the patient and any next of kin about immediate clinical care. It is often not possible for the

For peer review only

patient, relative or carer (consultee) to review trial documentation, consider the information and communicate an informed decision about whether they would wish to participate in the study. The consent procedure for this trial will reflect that of the surgery, with the clinical team assessing capacity before taking consent for the surgical procedure, and this capacity assessment then being used to guide the proper approach to consenting to the research. An appropriate method, in line with the Mental Capacity Act 2005 and the code of Practice 2007, and approved by the National Research Ethics Committee, will be used to gain either prospective or retrospective consent form the patient or appropriate consultee by a Good Clinical Practice (GCP)-trained, appropriately delegated member of the research team.

Post-randomisation withdrawals and exclusion

Participants/consultees may withdraw from the study at any time without prejudice. In addition, the investigator may discontinue a participant from the study at any time if the investigator considers it necessary. Throughout the study, screening logs will be kept to determine the number of patients assessed for eligibility and reasons for any exclusion.

If the participant/consultee withdraws from the study completely, data collected from the participant or recorded in the medical record up until the point of withdrawal will be included in the final analysis. Since randomisation will occur just prior to surgery, data regarding the operation received and autotransfusion blood volume (where deemed possible) will be recorded as a minimum for all participants. Participants who decline to continue to take part once they have regained capacity will be given the opportunity to discuss/inform the research team of the reasoning behind their decision not to take part.

Similarly, data from participants who die before consent to continue participating can be obtained, will be included in the final analysis. For those participants who lack capacity, and die before advice can be obtained from the participant's relatives/next of kin, it is our intention not to contact relatives of participants to inform them of the participant's initial inclusion in the study to avoid distressing the relatives unnecessarily.

Randomisation and blinding

The allocation sequence will be generated by the trial statistician. The treatment allocation will be on a 1:1 basis and will be stratified by fracture type (extracapsular vs intracapsular) and by trial centre, to ensure that any clustering effects within centres are evenly distributed between the treatment groups. The allocation will be administered using secure, online randomisation via a distant computer at Oxford Clinical Trials Research Unit (OCTRU), University of Oxford, using RRAMP software. Participants will be randomised pre-operatively. The research associate will inform the surgeon and the operating theatre staff of the allocation in the immediate pre-operative period.

For peer review only

In order to negate bias in the self-reported HRQoL outcome measures participants will be blinded to treatment allocation. The operating surgeon cannot be blinded to the allocation but they will not be involved in the assessment of outcomes. Patients will be blinded until the completion of the trial when the blinding will be broken if requested by the participants.

Treatments

Preoperative assessments

Diagnosis of a hip fracture will be confirmed by a plain radiograph, as per routine clinical care. Routine investigations, anaesthetic assessment, antibiotic and venous thromboembolic prophylaxis will be used as per local policy.

Anaesthetic technique

A regional or general anaesthesia technique will be used for every participant as per routine clinical care. Intra-operative analgesia may be achieved by combining a local anaesthetic nerve block, paracetamol and opiate analgesia as clinically indicated.

Trial Treatments

All participants will receive perioperative prophylactic antibiotics in accordance with current protocols agreed at each centre. Appropriate preparation, positioning and fracture reduction will be left to the discretion of the operating surgeon, as per their normal clinical practice. The need for allogenic blood products will be determined on an individual patient basis, following each centre's blood transfusion policy. This will typically involve restrictive transfusion thresholds where asymptomatic patients with a haemoglobin concentration of less than 70g/L are offered allogenic blood. This threshold may be higher, typically a haemoglobin concentration of less than 80g/L in those with symptomatic anaemia or coexisting cardiorespiratory disease.

Participants will be randomly allocated to one of the treatment arms:

Group 1: Standard Care

A standard suction system removes blood lost in the operating field and it is disposed of in clinical waste.

Group 2: Intra operative cell salvage and autotransfusion

Intraoperative cell salvage aspirates blood and lavage fluids from the operative field during surgery and returns it to the cell saver device where it is filtered and stored in an Anticoagulant Citrate Dextrose Solution. The recovered fluid will be washed with saline and centrifugated. In all cases where technically sufficient blood is available for transfusion, it will be transferred into a blood-giving bag, where the washed red blood cells, suspended in saline, will be transfused intraoperatively. The volume of blood that was transfused, when

For peer review only

this was possible, will be recorded. It will be the responsibility of the treating clinician to ensure this data is recorded in the clinical notes at the end of surgery. Other relevant information about the operation will be collected.

Postoperative rehabilitation

Postoperative analgesia will be prescribed intra-operatively and reviewed by the responsible clinical teams as appropriate. In the postoperative period, as per standard of care, all participants will undergo an initial physiotherapy and occupational therapy trauma assessment. As part of standard care, an initial treatment plan with objectives will be made, recorded and commenced. The aim of this plan will be for participants to mobilise through early, active, full weight bearing.

Participants will be discharged from the acute Orthopaedic Trauma Ward at the earliest safe opportunity to the most appropriate discharge destination as determined by the multi-disciplinary clinical team.

Outcomes

Personal data collected during the study will be handled and stored in accordance with the 2018 Data Protection Act, which requires data to be anonymised as soon as it is practical to do so. The data collected from participants will be entered in linked-anonymised form to the trial database. All electronic patient-identifiable information will be stored on a secure, password-protected database at the University of Oxford, accessible only to the research team.

Primary outcome Measure

The UK Core Outcome Set for hip fracture recommends that patient benefit is best determined by a measure of health-related quality-of-life.[30,31] The study primary outcome measure is EuroQol 5 Dimensions 5 Levels (EQ-5D-5L) score at 4-months post-injury. EQ-5D-5L is a validated instrument comprising a visual analogue scale (VAS) measuring self-rated health and a health status instrument, consisting of a five-level response (no problems, some problems, moderate problems, severe problems and unable) for five domains related to daily activities;[32,33] (i) mobility, (ii) self-care, (iii) usual activities, (iv) pain and discomfort and (v) anxiety and depression. Responses to the health status classification system will be converted into an overall score using a published utility algorithm for the UK population.[34] A respondent's EQ-VAS gives self-rated health on a scale where the endpoints are labelled 'best imaginable health state' (100) and 'worst imaginable health state' (0). It has been shown to be responsive to change,[31,35] including when reported by proxy for those with cognitive impairment.[36,37] Parsons et al[38] modelled patient EQ-5D recovery trajectories after hip fracture surgery to assess the extent of any bias in 4 months outcomes by comparing

complete case analysis, model-based projections and data imputation. They showed that imputing a utility of zero for death was a very close approximation to the much more complex projection methods, which was highly dependent on early (pre 4 months) EQ-5D score data that would not be available in the setting of a trial.[38]

Secondary outcome measures

Complications

All complications related to the index fracture and its treatment will be recorded. Complications will be classified as:

- related systemic complications[34] (including venous thromboembolic phenomena, death, pneumonia, urinary tract infection, blood transfusion, acute cerebrovascular incident, acute cardiac event, acute kidney injury, other).
- related local complications (superficial/deep infection, non/mal union, failure/removal/revision of metalwork including further surgery for intraoperative/ postoperative peri-prosthetic fracture, injury to adjacent structures such as nerves/tendons/blood vessels, other).
- unrelated to the trial protocol.

The number and type of related serious adverse events (SAEs) up to 12 months will be recorded.

Delirium

In line with data collection in the UK National Hip Fracture Database (NHFD) we will collect an immediate pre-operative abbreviated mental test score (AMTS) and a postoperative (up to 3 days) 4AT score.

Residential Status

Changes in residential status provide a marker for a participant's independence through their hip fracture recovery and is one of the recommended core outcomes for trials assessing interventions in hip fractures.[30] It will be reported by participants or their proxy using an ordinal scale as per the NHFD: (1) own home/sheltered housing, (2) residential care, (3) nursing care, (4) rehabilitation unit – hospital bed in the current trust, (5) rehabilitation unit – hospital bed in another trust, (6) rehabilitation unit – NHS funded care home bed, and (7) acute hospital.

Mobility

The ability to walk indoors and outdoors is rated very highly by patients.[30] Mobility will be reported by participants or their proxy using an ordinal scale as per the NHFD: (1) freely mobile without aids, (2) mobile outdoors with one aid, (3) mobile outdoors with two aids or

For peer review only - http://bmjopen.bmj.com/site/about/guidelines.xhtml

a frame, (4) some indoor mobility but never goes outside without help, and (5) no functional mobility using the lower limbs.

Units of allogenic blood transfused

The use of allogenic blood products during the index hospital stay will be collected from the trial centres' blood bank database. For each participant, the number of units transfused and the date of transfusion will be collected.

Mortality

Mortality during the first 12 months following surgery will be collected from NHS spine (NHS Digital; https://digital.nhs.uk/).

Resource use

Case report forms will be used to collect resources from medical records during the initial inpatient stay, and post discharge for 12 months at the treating hospital. Further resource use will be collected from the participants to complement the medical records. Participant questionnaires will be administered by telephone or post. They will enquire about hospital contacts related to the index fracture with hospitals other than the index treating sites, rehabilitation units and other care settings. Questions will also ask about the use of equipment and changes to the home, private expenses with rehabilitation services, informal care and loss of productivity.

Sample size

The sample size for this study is 1128 participants. This full trial sample size is based on the standard deviation of the EQ-5D-5L at 4 months post-surgery of 0.3 points[31] and a minimal clinically important difference of 0.075[39] with 2-sided significance of 5% requiring 506 with the primary outcome for 80% power or 676 with the primary outcome for 90% power.

In this population we expect considerable loss to follow-up. Previous WHiTE trials have indicated that these losses are due mainly to patients declining consent to further follow-up, incapacity, and death.[40,41] We are able to account for participants who have died in our primary outcome measure and have assumed that only 60% of recruited study participants will be available at the definitive endpoint at 4 months. With a significance level of 5%, this inflates the sample size to 844 for 80% power and 1128 for 90% power. Conservatively, we aim to randomise 1128 in order to ensure a minimum of 676 participants with the primary outcome which will ensure 90% power based upon these assumptions.

Similar sample size calculations have been used in existing clinical trials in this patient population (ISRCTN92825709, ISRCTN18393176).

Statistical analysis

A full, detailed Statistical Analysis Plan (SAP) will be drafted early in the trial and will be finalised following the recruitment review by the Data and Safety Monitoring Committee

For peer review only - http://bmjopen.bmj.com/site/about/guidelines.xhtml

(DSMC) and Trial Steering Committee (TSC), and prior to the primary analysis data lock. Any subsequent changes to the SAP will be fully justified in the final report.

Baseline demographic data will be summarised to check comparability between treatment arms. Standard statistical summaries and graphical plots will be presented for the primary outcome measure and all secondary outcome measures.

The study analysis will use generalised mixed-effects regression models, with all analyses adjusting for important baseline covariates to improve precision in estimation of the treatment effect. The principal analyses will be conducted on the intention-to-treat (ITT) population. Differences between intervention arms for the primary outcome measure, EQ-5D-5L[33] scores at four months post-surgery, will be analysed by calculating an adjusted treatment effect using a mixed-effects linear regression. A zero value will be imputed for participants who have died prior to this time point. Models will adjust for age, sex, fracture type and cognitive impairment (as fixed effects) and recruitment centre as a random effect to take account of the heterogeneity in the response between centres. The treatment difference will be estimated from the fitted model, together with 95% confidence intervals, with significance set at 5% (2-sided) for comparative tests.

A sensitivity analysis will be performed on a per-protocol (as treated) basis. Further sensitivity analysis of EQ-5D-5L[33] at 4 months with additional adjustment for the retrospective pre-injury baseline EQ-5D-5L[33] will be carried out to enable the influence of this factor to be evaluated.

Secondary clinical outcomes will be similarly analysed with logistic mixed-effects regression being used for binary data and linear mixed-effects regression for continuous data.

Adverse events will be explored to assess if they differ between groups.
Stata (StatCorp, LP) or other appropriate validated statistical software will be used for all analysis.

Cost-effectiveness analysis

A within-trial cost-effectiveness analysis will be conducted from the UK NHS and Personal Social Services perspective (PSS)[42] in the base case analysis. Resource utilisation involving cost of the cell salvage and autotransfusion if applicable will be obtained from case report forms (CRFs) that will be completed by the local research teams. Broader resource utilisation will be captured through CRFs and patient questionnaires administered at baseline, 4 months, and 12 months post-surgery. Unit costs for health and social care resources will largely be derived from the latest available local and national sources and estimated in line with best practice. Costs will be standardised to current prices where appropriate. An incremental cost-effectiveness analysis, expressed in terms of incremental cost per quality-

For peer review only

adjusted life year (QALY) gained, will be performed. Results will be presented using incremental cost-effectiveness ratios (ICERs), net monetary benefit, and cost effectiveness acceptability curves (CEACs) generated via non-parametric bootstrapping. Multiple imputation methods will be used to impute missing data and avoid biases associated with complete case analysis. Sensitivity analyses involving economic analysis from the societal perspective and extending the time frame from four months to one year will also be conducted.

Trial organisation and oversight

The sponsor of this trial is University Hospitals Coventry and Warwickshire NHS trust. The day-to-day management of the trial will be the responsibility of the trial manager, based at the University of Oxford and supported by OCTRU staff. This will be overseen by a trial management group, who will meet monthly to assess progress. It will be the responsibility of the trial manager to undertake training of the research associates at each of the study centres. The study statistician and health economist will be closely involved in setting up data capture systems, design of databases and clinical reporting forms.

A TSC and an independent DAMOCLES[43] compliant DSMC, that will assess progress, conduct and participant safety, will be set up at the start of the study.

Quality control

Quality control procedures will be undertaken during recruitment and data collection phases of the study to ensure research is conducted, generated, recorded and reported in compliance with the protocol, GCP and ethics committee. The chief investigators and the trial manager will develop data management and monitoring plans.

Patient and Public Involvement

At the centre of this trial is the potential for patient benefit by reducing the risks of hip fracture surgery and improving patient outcomes. The study proposal was discussed with our panel of 15 patient and public members. A member of this panel is a co-applicant on this trial and helped draft the protocol, lay summary and patient information sheet. A lay summary informing patients and the public of the trial outcome will be available on the trial website. Further documentation suitable for the general patient and public communities will be prepared by the research team in collaboration with lay representatives.

Ethics and Dissemination

This study will be embedded within the WHITE portfolio of trials. NHS ethical approval was provided on 14/08/2019 (19/WA/0197) and the trial registered (ISRCTN15945622). The results of this trial will be disseminated to the hip fracture clinical community via presentations at national and international meetings as well as publication in peer-reviewed journal. Results will be disseminated in lay form to participants and the public.

For peer review only

For peer review only - http://bmjopen.bmj.com/site/about/guidelines.xhtml

WORD COUNT 3977

DECLARATIONS

Ethics approval

Wales Research Ethics Committee 5 granted ethical approval for the study on 14th August 2019 (19/WA/0197). Participants or an appropriate consultee will provide consent/agreement to enter the study. The present version of the protocol is 5.0 15/7/2020.

Consent to publish

Not applicable

Availability of data and materials

Reasonable requests for access to the datasets can be made to Prof Xavier Griffin (X.griffin@qmul.ac.uk), three years after the publication of the clinical results of the study.

Funding

This project is funded by the National Institute for Health Research (NIHR), Research for Patient Benefit (PB-PG-0817-20037 and NIHR202013) and supported by the NIHR Oxford Biomedical Research Centre. The funder did/ will not have a role in study design, collection, analysis, interpretation or writing of the report.

Competing interests

XG is a NIHR Clinician Scientist. James M Mason was a member of the NIHR Health Services and Delivery Research Funding Committee.

Author Contributors

DG, XG, ED, JA, MW, MM, JM, DA, NP and JR were responsible for obtaining grant funding for this trial. NP and HO developed the statistical analysis plan and are leading the statistical analysis for the study. JM developed the economic analysis plan. AA and KM were responsible for administrative set up and performance of the trial. All authors developed the trial protocol and contributed to writing this manuscript. All authors reviewed and agreed the final manuscript.

Acknowledgements

Not applicable

Disclaimer

The views expressed in this report are those of the author(s) and not necessarily those of the NIHR, or the Department of Health and Social Care.

For peer review only

REFERENCES

1. National Hip Fracture Database annual report 2016 | RCP London. Accessed November 22, 2021. https://www.rcplondon.ac.uk/projects/outputs/national-hip-fracture-database-annual-report-2016
2. Gullberg B, Johnell O, Kanis JA. World-wide projections for hip fracture. *Osteoporosis international : a journal established as result of cooperation between the European Foundation for Osteoporosis and the National Osteoporosis Foundation of the USA*. 1997;7(5):407-413. doi:10.1007/PL00004148
3. Moran CG, Wenn RT, Sikand M, Taylor AM. Early mortality after hip fracture: is delay before surgery important? *The Journal of bone and joint surgery American volume*. 2005;87(3):483-489. doi:10.2106/JBJS.D.01796
4. Griffin XL, Parsons N, Achten J, Fernandez M, Costa ML. Recovery of health-related quality of life in a United Kingdom hip fracture population. The Warwick Hip Trauma Evaluation--a prospective cohort study. *The bone & joint journal*. 2015;97-B(3):372-382. doi:10.1302/0301-620X.97B3.35738
5. Kenzora JE, Mccarthy RE, J DRENNAN L, Sledge CB. Hip Fracture Mortality: Relation to Age, Treatment, Preopera... : Clinical Orthopaedics and Related Research®. *Clinical orthopaedics and related research 1984; 186: 45-56*. Published online 1984. Accessed November 22, 2021. https://journals.lww.com/clinorthop/Abstract/1984/06000/Hip_Fracture_Mortality__Relation_to_Age,.8.aspx
6. Roche JJW, Wenn RT, Sahota O, Moran CG. Effect of comorbidities and postoperative complications on mortality after hip fracture in elderly people: prospective observational cohort study. *BMJ (Clinical research ed)*. 2005;331(7529):1374-1376. doi:10.1136/BMJ.38643.663843.55
7. Kannegaard PN, van der Mark S, Eiken P, Abrahamsen B. Excess mortality in men compared with women following a hip fracture. National analysis of comedications, comorbidity and survival. *Age and ageing*. 2010;39(2):203-209. doi:10.1093/AGEING/AFP221
8. Penninx BWJH, Pahor M, Woodman RC, Guralnik JM. Anemia in old age is associated with increased mortality and hospitalization. *The journals of gerontology Series A, Biological sciences and medical sciences*. 2006;61(5):474-479. doi:10.1093/GERONA/61.5.474
9. Foss NB, Kehlet H. Hidden blood loss after surgery for hip fracture. *The Journal of bone and joint surgery British volume*. 2006;88(8):1053-1059. doi:10.1302/0301-620X.88B8.17534
10. Foss NB, Kristensen MT, Kehlet H. Anaemia impedes functional mobility after hip fracture surgery. *Age and ageing*. 2008;37(2):173-178. doi:10.1093/AGEING/AFM161

For peer review only - http://bmjopen.bmj.com/site/about/guidelines.xhtml

11. Penninx BWJH, Pluijm SMF, Lips P, et al. Late-life anemia is associated with increased risk of recurrent falls. *Journal of the American Geriatrics Society*. 2005;53(12):2106-2111. doi:10.1111/J.1532-5415.2005.00491.X
12. Brunskill SJ, Millette SL, Shokoohi A, et al. Red blood cell transfusion for people undergoing hip fracture surgery. *The Cochrane database of systematic reviews*. 2015;2015(4). doi:10.1002/14651858.CD009699.PUB2
13. Rohde JM, Dimcheff DE, Blumberg N, et al. Health care-associated infection after red blood cell transfusion: a systematic review and meta-analysis. *JAMA*. 2014;311(13):1317-1326. doi:10.1001/JAMA.2014.2726
14. Bou Monsef J, Boettner F. Blood management may have an impact on length of stay after total hip arthroplasty. *HSS journal : the musculoskeletal journal of Hospital for Special Surgery*. 2014;10(2):124-130. doi:10.1007/S11420-014-9384-X
15. Bolton-Maggs P, Poles D, Group eaobotSHoTSS. The 2016 annual SHOT Report, 2017. Accessed November 22, 2021. https://www.shotuk.org/wp-content/uploads/myimages/SHOT-Report-2016_web_11th-July.pdf
16. Excellence NIoHaC. Costing Statement | Blood transfusion | Guidance | NICE. Published 2015. Accessed November 22, 2021. https://www.nice.org.uk/guidance/NG24/resources
17. Excellence NIoHaC. Blood transfusion | Guidance | NICE. Published 2015. Accessed November 22, 2021. https://www.nice.org.uk/guidance/ng24
18. Parker MJ, Gurusamy KS. Internal fixation versus arthroplasty for intracapsular proximal femoral fractures in adults. *The Cochrane database of systematic reviews*. 2006;2006(4). doi:10.1002/14651858.CD001708.PUB2
19. Parker MJ, Handoll HHG. Replacement arthroplasty versus internal fixation for extracapsular hip fractures in adults. *The Cochrane database of systematic reviews*. 2006;2006(2). doi:10.1002/14651858.CD000086.PUB2
20. Queally JM, Harris E, Handoll HHG, Parker MJ. Intramedullary nails for extracapsular hip fractures in adults. *The Cochrane database of systematic reviews*. 2014;2014(9). doi:10.1002/14651858.CD004961.PUB4
21. Parker MJ, Das A. Extramedullary fixation implants and external fixators for extracapsular hip fractures in adults. *The Cochrane database of systematic reviews*. 2013;2013(2). doi:10.1002/14651858.CD000339.PUB3
22. Kim SY, Kim YG, Hwang JK. Cementless calcar-replacement hemiarthroplasty compared with intramedullary fixation of unstable intertrochanteric fractures. A prospective, randomized study. *The Journal of bone and joint surgery American volume*. 2005;87(10):2186-2192. doi:10.2106/JBJS.D.02768
23. Vossinakis IC, Badras LS. The external fixator compared with the sliding hip screw for pertrochanteric fractures of the femur. *The Journal of bone and joint surgery British volume*. 2002;84(1):23-29. doi:10.1302/0301-620X.84B1.12034

For peer review only

24. Rödén M, Schön M, Fredin H. Treatment of displaced femoral neck fractures: a randomized minimum 5-year follow-up study of screws and bipolar hemiprostheses in 100 patients. *Acta orthopaedica Scandinavica*. 2003;74(1):42-44. doi:10.1080/00016470310013635

25. Tidermark J, Ponzer S, Svensson O, Söderqvist A, Törnkvist H. Internal fixation compared with total hip replacement for displaced femoral neck fractures in the elderly. A randomised, controlled trial. *The Journal of bone and joint surgery British volume*. 2003;85(3):380-388. doi:10.1302/0301-620X.85B3.13609

26. Costa ML, Griffin XL, Achten J, et al. World Hip Trauma Evaluation (WHiTE): framework for embedded comprehensive cohort studies. *BMJ open*. 2016;6(10). doi:10.1136/BMJOPEN-2016-011679

27. Griffin XL, McArthur J, Achten J, Parsons N, Costa ML. The Warwick Hip Trauma Evaluation One -an abridged protocol for the WHiTE One Study: An embedded randomised trial comparing the X-bolt with slidinghip screw fixation in extracapsular hip fractures. *Bone & joint research*. 2013;2(10):206-209. doi:10.1302/2046-3758.210.2000183

28. Huxley C, Achten J, Costa ML, Griffiths F, Griffin XL. A process evaluation of the WHiTE Two trial comparing total hip arthroplasty with and without dual mobility component in the treatment of displaced intracapsular fractures of the proximal femur: Can a trial investigating total hip arthroplasty for hip fracture be delivered in the NHS? *Bone & joint research*. 2016;5(10):444-452. doi:10.1302/2046-3758.510.BJR-2015-0008.R1

29. Sims AL, Parsons N, Achten J, Griffin XL, Costa ML, Reed MR. The World Hip Trauma Evaluation Study 3: Hemiarthroplasty Evaluation by Multicentre Investigation - WHITE 3: HEMI - An Abridged Protocol. *Bone & joint research*. 2016;5(1):18-25. doi:10.1302/2046-3758.51.2000473

30. Haywood KL, Griffin XL, Achten J, Costa ML. Developing a core outcome set for hip fracture trials. *The bone & joint journal*. 2014;96-B(8):1016-1023. doi:10.1302/0301-620X.96B8.33766

31. Parsons N, Griffin XL, Achten J, Costa ML. Outcome assessment after hip fracture: is EQ-5D the answer? *Bone & joint research*. 2014;3(3):69-75. doi:10.1302/2046-3758.33.2000250

32. Brooks R, de Charro F. EuroQol: the current state of play. *Health policy (Amsterdam, Netherlands)*. 1996;37(1):53-72. doi:10.1016/0168-8510(96)00822-6

33. Herdman M, Gudex C, Lloyd A, et al. Development and preliminary testing of the new five-level version of EQ-5D (EQ-5D-5L). *Quality of life research : an international journal of quality of life aspects of treatment, care and rehabilitation*. 2011;20(10):1727-1736. doi:10.1007/S11136-011-9903-X

34. Dolan P. Modeling valuations for EuroQol health states. *Medical care*. 1997;35(11):1095-1108. doi:10.1097/00005650-199711000-00002

For peer review only

35. Tidermark J, Bergström G. Responsiveness of the EuroQol (EQ-5D) and the Nottingham Health Profile (NHP) in elderly patients with femoral neck fractures. *Quality of life research : an international journal of quality of life aspects of treatment, care and rehabilitation*. 2007;16(2):321-330. doi:10.1007/S11136-006-9004-4

36. Bryan S, Hardyman W, Bentham P, Buckley A, Laight A. Proxy completion of EQ-5D in patients with dementia. *Quality of life research : an international journal of quality of life aspects of treatment, care and rehabilitation*. 2005;14(1):107-118. doi:10.1007/S11136-004-1920-6

37. Hounsome N, Orrell M, Edwards RT. EQ-5D as a quality of life measure in people with dementia and their carers: evidence and key issues. *Value in health : the journal of the International Society for Pharmacoeconomics and Outcomes Research*. 2011;14(2):390-399. doi:10.1016/J.JVAL.2010.08.002

38. Parsons N, Griffin XL, Achten J, Chesser TJ, Lamb SE, Costa ML. Modelling and estimation of health-related quality of life after hip fracture: A re-analysis of data from a prospective cohort study. *Bone & joint research*. 2018;7(1):1-5. doi:10.1302/2046-3758.71.BJR-2017-0199

39. Walters SJ, Brazier JE. Comparison of the minimally important difference for two health state utility measures: EQ-5D and SF-6D. *Quality of life research : an international journal of quality of life aspects of treatment, care and rehabilitation*. 2005;14(6):1523-1532. doi:10.1007/S11136-004-7713-0

40. Sims AL, Parsons N, Achten J, Griffin XL, Costa ML, Reed MR. A randomized controlled trial comparing the Thompson hemiarthroplasty with the Exeter polished tapered stem and Unitrax modular head in the treatment of displaced intracapsular fractures of the hip: the WHiTE 3: HEMI Trial. *The bone & joint journal*. 2018;100-B(3):352-360. doi:10.1302/0301-620X.100B3.BJJ-2017-0872.R2

41. Griffin XL, Achten J, Sones W, Cook J, Costa ML. Randomised controlled trial of the sliding hip screw versus X-Bolt Dynamic Hip Plating System for the fixation of trochanteric fractures of the hip in adults: a protocol study for WHiTE 4 (WHiTE4). *BMJ open*. 2018;8(1). doi:10.1136/BMJOPEN-2017-019944

42. Guide to the Methods of Technology Appraisal 2013. London. Accessed November 22, 2021. https://pubmed.ncbi.nlm.nih.gov/27905712/

43. A proposed charter for clinical trial data monitoring committees: helping them to do their job well. *Lancet (London, England)*. 2005;365(9460):711-722. doi:10.1016/S0140-6736(05)17965-3

For peer review only

SPIRIT — Standard Protocol Items: Recommendations for Interventional Trials

SPIRIT 2013 Checklist: Recommended items to address in a clinical trial protocol and related documents*

Section/item	Item No	Description	Page/ Location
**Administrative information**			
Title	1	Descriptive title identifying the study design, population, interventions, and, if applicable, trial acronym	1
Trial registration	2a	Trial identifier and registry name. If not yet registered, name of intended registry	2
	2b	All items from the World Health Organization Trial Registration Data Set	2
Protocol version	3	Date and version identifier	15
Funding	4	Sources and types of financial, material, and other support	16
Roles and responsibilities	5a	Names, affiliations, and roles of protocol contributors	16
	5b	Name and contact information for the trial sponsor	14
	5c	Role of study sponsor and funders, if any, in study design; collection, management, analysis, and interpretation of data; writing of the report; and the decision to submit the report for publication, including whether they will have ultimate authority over any of these activities	16
	5d	Composition, roles, and responsibilities of the coordinating centre, steering committee, endpoint adjudication committee, data management team, and other individuals or groups overseeing the trial, if applicable (see Item 21a for data monitoring committee)	14

For peer review only

**Introduction**			
Background and rationale	6a	Description of research question and justification for undertaking the trial, including summary of relevant studies (published and unpublished) examining benefits and harms for each intervention	4
	6b	Explanation for choice of comparators	4
Objectives	7	Specific objectives or hypotheses	6
Trial design	8	Description of trial design including type of trial (eg, parallel group, crossover, factorial, single group), allocation ratio, and framework (eg, superiority, equivalence, noninferiority, exploratory)	7
**Methods: Participants, interventions, and outcomes**			
Study setting	9	Description of study settings (eg, community clinic, academic hospital) and list of countries where data will be collected. Reference to where list of study sites can be obtained	7
Eligibility criteria	10	Inclusion and exclusion criteria for participants. If applicable, eligibility criteria for study centres and individuals who will perform the interventions (eg, surgeons, psychotherapists)	7
Interventions	11a	Interventions for each group with sufficient detail to allow replication, including how and when they will be administered	9
	11b	Criteria for discontinuing or modifying allocated interventions for a given trial participant (eg, drug dose change in response to harms, participant request, or improving/worsening disease)	7-9
	11c	Strategies to improve adherence to intervention protocols, and any procedures for monitoring adherence (eg, drug tablet return, laboratory tests)	7-9
	11d	Relevant concomitant care and interventions that are permitted or prohibited during the trial	9

For peer review only - http://bmjopen.bmj.com/site/about/guidelines.xhtml

Outcomes	12	Primary, secondary, and other outcomes, including the specific measurement variable (eg, systolic blood pressure), analysis metric (eg, change from baseline, final value, time to event), method of aggregation (eg, median, proportion), and time point for each outcome. Explanation of the clinical relevance of chosen efficacy and harm outcomes is strongly recommended	10-12
Participant timeline	13	Time schedule of enrolment, interventions (including any run-ins and washouts), assessments, and visits for participants. A schematic diagram is highly recommended (see Figure)	7-12
Sample size	14	Estimated number of participants needed to achieve study objectives and how it was determined, including clinical and statistical assumptions supporting any sample size calculations	12
Recruitment	15	Strategies for achieving adequate participant enrolment to reach target sample size	7-8
**Methods: Assignment of interventions (for controlled trials)**			
Allocation:			
Sequence generation	16a	Method of generating the allocation sequence (eg, computer-generated random numbers), and list of any factors for stratification. To reduce predictability of a random sequence, details of any planned restriction (eg, blocking) should be provided in a separate document that is unavailable to those who enrol participants or assign interventions	8-9
Allocation concealment mechanism	16b	Mechanism of implementing the allocation sequence (eg, central telephone; sequentially numbered, opaque, sealed envelopes), describing any steps to conceal the sequence until interventions are assigned	8-9
Implementation	16c	Who will generate the allocation sequence, who will enrol participants, and who will assign participants to interventions	8-9
Blinding (masking)	17a	Who will be blinded after assignment to interventions (eg, trial participants, care providers, outcome assessors, data analysts), and how	9

	17b	If blinded, circumstances under which unblinding is permissible, and procedure for revealing a participant's allocated intervention during the trial	9
**Methods: Data collection, management, and analysis**			
Data collection methods	18a	Plans for assessment and collection of outcome, baseline, and other trial data, including any related processes to promote data quality (eg, duplicate measurements, training of assessors) and a description of study instruments (eg, questionnaires, laboratory tests) along with their reliability and validity, if known. Reference to where data collection forms can be found, if not in the protocol	10-12
	18b	Plans to promote participant retention and complete follow-up, including list of any outcome data to be collected for participants who discontinue or deviate from intervention protocols	7-8
Data management	19	Plans for data entry, coding, security, and storage, including any related processes to promote data quality (eg, double data entry; range checks for data values). Reference to where details of data management procedures can be found, if not in the protocol	10
Statistical methods	20a	Statistical methods for analysing primary and secondary outcomes. Reference to where other details of the statistical analysis plan can be found, if not in the protocol	12-14
	20b	Methods for any additional analyses (eg, subgroup and adjusted analyses)	12-14
	20c	Definition of analysis population relating to protocol non-adherence (eg, as randomised analysis), and any statistical methods to handle missing data (eg, multiple imputation)	12-14
**Methods: Monitoring**			
Data monitoring	21a	Composition of data monitoring committee (DMC); summary of its role and reporting structure; statement of whether it is independent from the sponsor and competing interests; and reference to where further details about its charter can be found, if not in the protocol. Alternatively, an explanation of why a DMC is not needed	14

For peer review only - http://bmjopen.bmj.com/site/about/guidelines.xhtml

	21b	Description of any interim analyses and stopping guidelines, including who will have access to these interim results and make the final decision to terminate the trial	12-14
Harms	22	Plans for collecting, assessing, reporting, and managing solicited and spontaneously reported adverse events and other unintended effects of trial interventions or trial conduct	11
Auditing	23	Frequency and procedures for auditing trial conduct, if any, and whether the process will be independent from investigators and the sponsor	14
**Ethics and dissemination**			
Research ethics approval	24	Plans for seeking research ethics committee/institutional review board (REC/IRB) approval	15
Protocol amendments	25	Plans for communicating important protocol modifications (eg, changes to eligibility criteria, outcomes, analyses) to relevant parties (eg, investigators, REC/IRBs, trial participants, trial registries, journals, regulators)	15
Consent or assent	26a	Who will obtain informed consent or assent from potential trial participants or authorised surrogates, and how (see Item 32)	7-8
	26b	Additional consent provisions for collection and use of participant data and biological specimens in ancillary studies, if applicable	n/a
Confidentiality	27	How personal information about potential and enrolled participants will be collected, shared, and maintained in order to protect confidentiality before, during, and after the trial	10
Declaration of interests	28	Financial and other competing interests for principal investigators for the overall trial and each study site	16
Access to data	29	Statement of who will have access to the final trial dataset, and disclosure of contractual agreements that limit such access for investigators	12

For peer review only - http://bmjopen.bmj.com/site/about/guidelines.xhtml

Ancillary and post-trial care	30	Provisions, if any, for ancillary and post-trial care, and for compensation to those who suffer harm from trial participation	n/a
Dissemination policy	31a	Plans for investigators and sponsor to communicate trial results to participants, healthcare professionals, the public, and other relevant groups (eg, via publication, reporting in results databases, or other data sharing arrangements), including any publication restrictions	14
	31b	Authorship eligibility guidelines and any intended use of professional writers	16
	31c	Plans, if any, for granting public access to the full protocol, participant-level dataset, and statistical code	12
**Appendices**			
Informed consent materials	32	Model consent form and other related documentation given to participants and authorised surrogates	n/a
Biological specimens	33	Plans for collection, laboratory evaluation, and storage of biological specimens for genetic or molecular analysis in the current trial and for future use in ancillary studies, if applicable	n/a

*It is strongly recommended that this checklist be read in conjunction with the SPIRIT 2013 Explanation & Elaboration for important clarification on the items. Amendments to the protocol should be tracked and dated. The SPIRIT checklist is copyrighted by the SPIRIT Group under the Creative Commons "Attribution-NonCommercial-NoDerivs 3.0 Unported" license.

For peer review only
