# Supplementary material for: Randomised controlled trial comparing intraoperative cell salvage and autotransfusion with standard care in the treatment of hip fractures: a protocol for the WHITE 9 study
Source: BMJ Open. 2022 Jun 8;12(6):e062338. doi: 10.1136/bmjopen-2022-062338 (PMC9185657; doi:10.1136/bmjopen-2022-062338)
Supplement: Reviewer comments [file bmjopen-2022-062338.reviewer_comments.pdf]

PEER REVIEW HISTORY

BMJ Open publishes all reviews undertaken for accepted manuscripts. Reviewers are asked to complete a checklist review form (**http://bmjopen.bmj.com/site/about/resources/checklist.pdf**) and are provided with free text boxes to elaborate on their assessment. These free text comments are reproduced below.

ARTICLE DETAILS

**TITLE (PROVISIONAL)**	A randomised controlled trial comparing intra-operative cell salvage and autotransfusion with standard care in the treatment of hip fractures: a protocol for the WHITE 9 study
**AUTHORS**	Dickenson, Edward; Griffin, Xavier; Achten, Juul; Mironov, Katy; O'Connor, Heather; Parsons, Nicholas; Murphy, Mike; Wyse, Matthew; Mason, James; Appelbe, Duncan; Athwal, Amrita; Griffin, Damian

This article was not externally reviewed at BMJ Open. Protocols that have been independently assessed prior to submission to BMJ Open are usually fast-tracked to publication on the grounds that further substantial changes will not be possible. This independent assessment will usually be external, independent review for both a substantial grant award from a non-commercial or government funder and ethics approval.
